# Genome-wide histone state profiling of fibroblasts from the opossum, *Monodelphis domestica*, identifies the first marsupial-specific imprinted gene

**DOI:** 10.1186/1471-2164-15-89

**Published:** 2014-01-31

**Authors:** Kory C Douglas, Xu Wang, Madhuri Jasti, Abigail Wolff, John L VandeBerg, Andrew G Clark, Paul B Samollow

**Affiliations:** 1 Department of Veterinary Integrative Biosciences, Texas A&M University, College Station, TX 77843, USA; 2 Department of Molecular Biology & Genetics, Cornell University, Ithaca, NY 14853, USA; 3 The Cornell Center for Comparative and Population Genomics, Cornell University, Ithaca, NY 14853, USA; 4 Department of Genetics, Texas Biomedical Research Institute, and Southwest National Primate Research Center, San Antonio, TX 78245, USA

**Keywords:** Genomic imprinting, Monoallelic expression, Histone modification, ChIP-seq, *Monodelphis domestica*, Marsupial

## Abstract

**Background:**

Imprinted genes have been extensively documented in eutherian mammals and found to exhibit significant interspecific variation in the suites of genes that are imprinted and in their regulation between tissues and developmental stages. Much less is known about imprinted loci in metatherian (marsupial) mammals, wherein studies have been limited to a small number of genes previously known to be imprinted in eutherians. We describe the first *ab initio* search for imprinted marsupial genes, in fibroblasts from the opossum, *Monodelphis domestica*, based on a genome-wide ChIP-seq strategy to identify promoters that are simultaneously marked by mutually exclusive, transcriptionally opposing histone modifications.

**Results:**

We identified a novel imprinted gene (*Meis1*) and two additional monoallelically expressed genes, one of which (*Cstb*) showed allele-specific, but non-imprinted expression. Imprinted vs. allele-specific expression could not be resolved for the third monoallelically expressed gene (*Rpl17*). Transcriptionally opposing histone modifications H3K4me3, H3K9Ac, and H3K9me3 were found at the promoters of all three genes, but differential DNA methylation was not detected at CpG islands at any of these promoters.

**Conclusions:**

In generating the first genome-wide histone modification profiles for a marsupial, we identified the first gene that is imprinted in a marsupial but not in eutherian mammals. This outcome demonstrates the practicality of an *ab initio* discovery strategy and implicates histone modification, but not differential DNA methylation, as a conserved mechanism for marking imprinted genes in all therian mammals. Our findings suggest that marsupials use multiple epigenetic mechanisms for imprinting and support the concept that lineage-specific selective forces can produce sets of imprinted genes that differ between metatherian and eutherian lines.

## Background

Genomic imprinting is an epigenetic phenomenon resulting in highly skewed (monoallelic) expression of genes in a parent-of-origin-specific manner. It affects a minority of genes in the genomes of therian mammals (eutherians and metatherians) but has not been detected in prototherians, birds, or other vertebrates [1-3]. In human and mouse, 79 and 123 imprinted genes have been characterized, respectively, with only ~60% of these genes sharing imprinted status in both species [4]. In addition to interspecific differences, imprinted expression of some loci has been shown to vary between cell types, tissues, developmental stages, and gene isoforms; and in some cases, ‘leaky’ expression of the repressed allele has been observed, especially in placenta [5-10]. These variable characteristics compound the difficulty of finding and describing imprinted genes, reveal the magnitude of variation present among suites of imprinted genes between species, and underscore the dynamic expression patterns of imprinted genes within an individual.

In metatherian (a.k.a. marsupial) mammals, genomic imprinting has been examined primarily in the tammar wallaby (*Macropus eugenii*: Australasian family Macropodidae), gray short-tailed opossum (*Monodelphis domestica*: American family Didelphidae), and Virginia opossum (*Didelphis virginiana*: Didelphidae), wherein only 19 genes, each already known to be imprinted in human and/or mouse, have been scrutinized in one or another of these species with regard to parent-of-origin-specific allele expression. Eight of these 19 loci have been shown to be imprinted in at least one of these marsupial species; nine show biallelic expression; and two have no marsupial homolog [11-14]. Of the eight marsupial imprinted genes, only *IGF2* and *H19* are located in an imprinted cluster and associated with an imprinting control region (ICR), both of which are hallmarks of imprinted loci in eutherian mammals [15,16]. The remaining six marsupial imprinted genes are individually imprinted, associated with no known clusters, and mechanisms that regulate their expression remain unknown.

Beyond interspecific comparative analyses to infer the evolutionary origins and adaptive significance of imprinted genes, the process of genomic imprinting is, *per se*, an invaluable model system for studying the epigenetic regulation of genes generally. For example, interspecific comparisons of imprinted and non-imprinted orthologs have led to the identification of certain structural features, such as SINEs and LINEs and their *cis*-acting epigenetic elements, that can affect the imprintability of a gene [17-19]. Further, the identification of differential DNA methylation between the two parental alleles at imprinted loci in eutherians has not only provided insight concerning the epigenetic regulation of these loci, but has also led to the development of a paradigm for studying *cis*-acting mechanisms of gene regulation at non-imprinted loci [3]. Finally, the interaction of genomic elements and epigenetic modifications at imprinted loci has revealed links between epigenetic states, chromatin structure, and transcriptional activity. A comprehensive catalogue of imprinted loci across a broader range of therians, including eutherian and marsupial species alike, with descriptions of the molecular mechanisms that establish and maintain the imprinted state, can illuminate the evolutionary history and mechanisms of genomic imprinting generally and perhaps reveal heretofore unrecognized selective pressures that act on a gene to target it for imprinted expression.

Various epigenetic marks have been associated with imprinted genes and ICRs in eutherians, most notably cytosine methylation and histone modifications. Differential methylation of cytosine residues at CpG dinucleotides within CpG islands has been found at both ICRs and promoter regions of imprinted genes and occurs in a parent-of-origin-allele-specific manner [20,21]. Some of these parent-of-origin-specific differentially methylated regions (DMRs) are established in the germ-line and maintained throughout all developmental stages and tissues, whereas other DMRs arise after fertilization and occur in tissue-specific or developmental-stage-specific patterns [22,23]. Furthermore, the loss of DNA methylation at the promoter region or ICR of an imprinted gene or imprinted gene cluster leads to the loss of the imprinted state, resulting in biallelic expression [24-26].

Differential histone modification states have also been associated with ICRs and promoter regions of imprinted genes. Transcriptionally repressive modifications such as trimethylation of lysine 9 of histone 3 (H3K9me3) and trimethylation of lysine 27 of histone 3 (H3K27me3) are present at the ICRs and/or promoters of the repressed allele, whereas transcriptionally active marks such as trimethylation of lysine 4 of histone 3 (H3K4me3) and acetylation of lysine 9 of histone 3 (H3K9Ac) are present at the ICRs and promoters of the actively expressed allele [27-29]. Along with DNA methylation, these histone modifications create relatively open or closed chromatin states, which can alter the accessibility of DNA to transcriptional machinery, thereby affecting transcription rates. In addition, certain of these transcriptionally opposing histone modifications have been shown to be mutually exclusive at identical sites in the promoter regions of active vs. repressed alleles at imprinted loci (e.g. H3K4me3 vs. H3K9me3; H3K9Ac vs. H3K9me3) suggesting a potentially powerful approach for seeking candidate-imprinted loci, independent of expression-based analyses based on DNA sequence variation, such as single-nucleotide polymorphisms (SNPs) [12,28,30].

Previous searches for imprinted genes in marsupials have focused on a small number of loci that are already known to be imprinted in eutherians, and only a few of these have sought to describe DNA methylation and histone modification profiles of CpG islands at putative promoter regions [2,13-15,31-37]. Clear evidence of differential DNA methylation at marsupial imprinted genes has been found at only two loci, and only the DMR at the *IGF2*-*H19* imprinting cluster has been shown to regulate transcription of a marsupial imprinted gene [15,33]. In addition, the marsupial X chromosome, which exhibits paternal imprinting for most loci in females, is strongly deficient in CpG island methylation [38-40].

Data addressing histone modifications at promoters and CpG islands of marsupial imprinted genes are extremely limited, with only two histone modifications, H3K3me2 and H3K9me3, examined for opossum *Igf2r*, *Htr2A*, and *L3mbtl*. These genes exhibit enrichment of H3K4me2 but not H3K9me3 at promoters [13]. Chromosome-level immunofluorescence analyses of wallaby, opossum, and brush-tailed possum (*Trichosurus vulpecula*: Australasian family Phalangeridae) X chromosomes, using antibodies to specific histone modifications, have shown correlations between several repressive and activating histone marks on the inactive and active X chromosomes, respectively, consistent with a possible role for histone modification states in the transcriptional regulation of genes on the marsupial X [39,41-44]. Unfortunately, these cytologic approaches lack power to resolve the locations of modified histones on scales much below the chromosome band level, so cannot identify correlations between histone modification distributions and expression states of individual genes.

Taking advantage of continuously improving next-generation (NextGen) sequencing technologies and the high-quality draft assembly of the *M. domestica* genome, we are now able to search for marsupial-specific imprinted genes and analyze fundamental signals of imprinting on a genome-wide basis. To accomplish this, we conducted reciprocal crosses of animals from two *M. domestica* stocks and used ChIP-seq (chromatin-immunoprecipitation of genomic DNA followed by NextGen sequencing) to perform the first *ab initio* search for putative gene promoters that are concurrently marked by mutually exclusive, transcriptionally opposing histone modifications as a means to identify candidate-imprinted genes.

## Results

### ChIP-seq analysis

**Figure 1:**
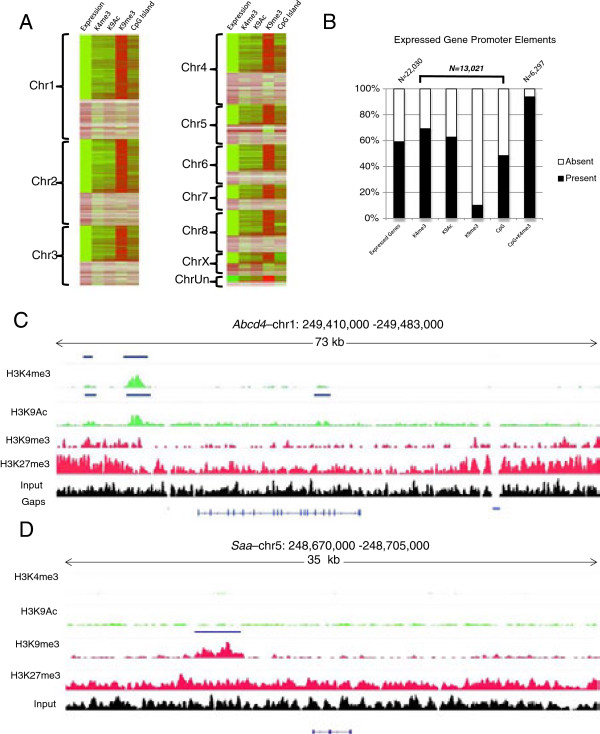
**Summary of fibroblast ChIP**-**seq results. A)** Heatmap of RNA expression, H3K4me3, H3K9Ac, and H3K9me3 occupancy, and CpG Islands. Expression was determined using RNA-seq data from four male fibroblast cell lines. Green = expression or presence of element, Red = no expression or absence of element. White = no measurable expression or elements. **B)** Graph of percentage of genes that are expressed, percentage of promoters of expressed genes with indicated histone modifications or CpG island, and percentage of CpG islands marked by H3K4me3. K4me3 = H3K4me3; K9Ac = H3K9Ac; K9me3 = H3K9me3. **C)** and **D)** Histone profiles across an expressed gene (*Abcd4*) and a repressed gene (*Saa*). Blue bars = significant peak of enrichment within 5.5 kb of the putative promoter; green scans = marks of activation; red scans = marks of repression; black scans = input. Gaps (if present) and gene annotations are shown in the bottom panels.

The genomic distributions of four histone modifications were analyzed in opossum fibroblasts by ChIP-seq, using antibodies against H3K4me3, H3K9me3, H3K27me3, and H3K9Ac (see Methods). More than 436 million Illumina ChIP-seq reads from male fibroblasts were uniquely mapped to the current *M. domestica* genome assembly (MonDom5). The two marks of activation (MOAs) examined, H3K4me3 and H3K9Ac, gave 79,412 and 52,511 unique peaks of enrichment, respectively (Model-based Analysis for Chip-seq [MACS], p ≤ 10^-5^, see Methods). The two marks of repression (MORs) examined, H3K9me3 and H3K27me3, gave 56,719 and 16,592 unique peaks of enrichment, respectively (MACS, p ≤ 10^-5^) (Additional file 1: Table S1). We next analyzed the overlap of each histone modification with promoters of annotated genes (defined herein as genomic regions 5,000 bases upstream to 500 bases downstream of annotated transcription start sites) and their associated CpG islands. Of the 22,030 annotated genes in MonDom5, 13,021 showed expression in at least one of four male-fibroblast cell lines as determined by RNA-seq (data not shown), and 9,012 (69%) of them were marked by H3K4me3 (Figure 1A, B). About half of these expressed genes have an annotated CpG island at the promoter and 93% of these CpG islands were marked with H3K4me3 regardless of transcriptional state (Figure 1B). Thus, the promoters of the transcribed genes (e.g. *Abcd4*, Figure 1C) showed enrichment for two MOAs and were deficient for MORs, whereas the promoters of repressed genes (e.g. *Saa*, Figure 1D) showed a deficiency in MOAs and, in some cases, an enrichment of H3K9me3. The distribution of H3K27me3 was diffuse across the opossum genome. Most significant peaks occurred in intergenic regions, while promoters and gene bodies of biallelically expressed genes and known opossum imprinted genes showed a general depletion of H3K27me3. In addition, enrichment of H3K27me3 has not been shown in other mammalian species to be mutually exclusive with the MOAs used in this study. For these reasons H3K27me3 appeared not to be useful for the purposes of this study and was excluded from further examination.

**Figure 2:**
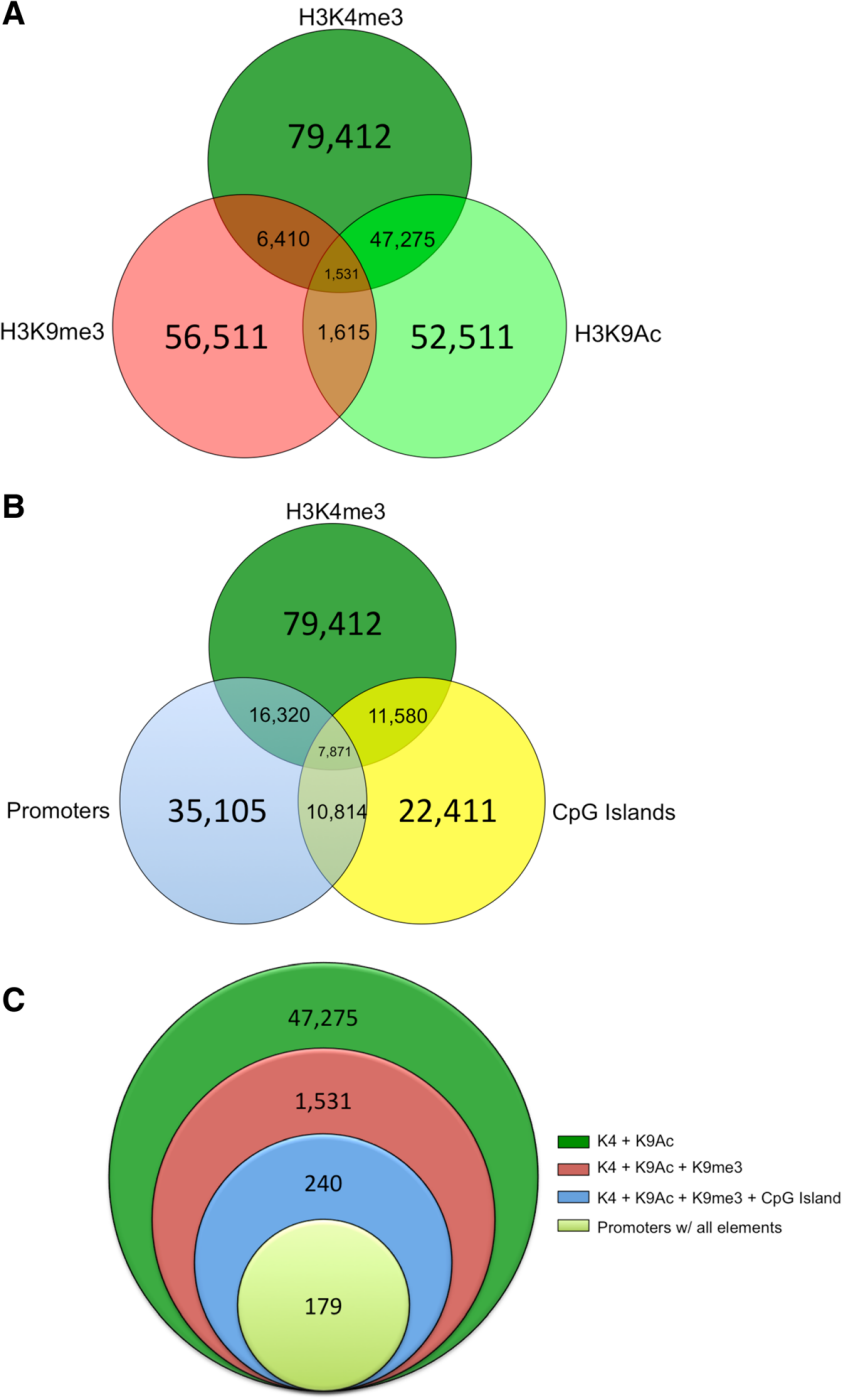
**Venn diagrams representing overlaps of significant histone peaks, annotated CpG islands, and putative promoters used to identify candidate-imprinted genes. A)** Significant peaks for H3K4me3 (dark green), H3K9Ac (light green) and H3K9me3 (red). **B)** Overlaps of H3K4me3 (green), annotated CpG islands (yellow), and putative promoters (blue). **C)** Overlaps of H3K4me3 + H3K9Ac peaks (green), all three histone modification peaks (red), all three histone modification peaks and CpG islands (blue), and all elements and putative promoters which represents the list of candidate imprinted genes (light green).

In addition to the promoters discussed above, we examined overlap of the various histone modifications with each other and all annotated putative promoters (35,105) in the MonDom5 assembly. Of the H3K9Ac peaks, 47,275 (90%) overlapped with an H3K4me3 peak by at least one base pair, and 6,410 (11%) H3K9me3 peaks overlapped with an H3K4me3 peak (Figure 2A, Additional file 1: Table S2). Additionally, 11,580 (52%) promoter-associated CpG islands were marked by a significant H3K4me3 peak.

Of the 35,105 putative promoters, 16,620 (~46%) were marked with H3K4me3, 7,871 also had an annotated CpG island, and 179 of them were also concurrently marked with H3K9Ac and H3K9me3 (Figure 2B and C). No X-linked genes met these criteria. This is noteworthy because the fibroblasts analyzed were of male origin (see Methods), and thus possessed only a single X chromosome; patterns reflective of imprinted expression of X-linked genes would have signaled false positive outcomes. That none were observed provides an internal control indicating the accuracy of ChIP-seq procedures in this study. These 179 autosomal genes with putative promoters marked by two MOAs, one MOR (H3K9me3), and a CpG island were considered candidate-imprinted genes and targeted for SNP discovery along with *Igf2r* (Figure 2C). *Igf2r* is known to be imprinted in *M. domestica* and has a promoter CpG island, but it did not show overlapping enrichment of MOAs and H3K9me3. The histone modification states of the remaining annotated opossum imprinted genes, *Htr2A*, *L3mbtl*, and *Mest*, were also examined and showed the presence of MOAs at their promoters but lacked H3K9me3 (Additional file 2: Figure S5). However, informative SNPs for these genes were not present in our crosses, precluding our assessment of their imprinted/non-imprinted states. Furthermore, the *Igf2*-*H19* imprinted cluster is not present in the current MonDom5 assembly and consequently was not accessible for this study.

### SNP search for candidate-imprinted genes

PCR primers (Additional file 1: Table S3) were designed to target the 3’-UTR for each of the 179 candidate-imprinted genes and *Igf2r*, allowing for amplification of both genomic DNA (gDNA) and cDNA with the same primer. The primer panel was run on liver DNA from the eight animals in the P generation to search for ‘trackable’ parent-specific SNPs between the reciprocal crosses. Of the 179 genes tested, 38–49 genes, depending on the cross, showed at least one such SNP in individual crosses (Additional file 1: Tables S4-S7). We selected 30 genes that had a trackable SNP in at least one family in each reciprocal cross, and 21 of these showed specific 3’-UTR amplification of cDNA from the F_1_ generation. PCR products amplified from fibroblast-derived gDNA and cDNA using these primers were Sanger sequenced to qualitatively assess monoallelic vs. biallelic gene expression, and 17 of them gave high quality sequences from both templates (Additional file 1: Tables S4-S7).

### *Meis1* is paternally imprinted in *M. domestica* fibroblasts

**Figure 3:**
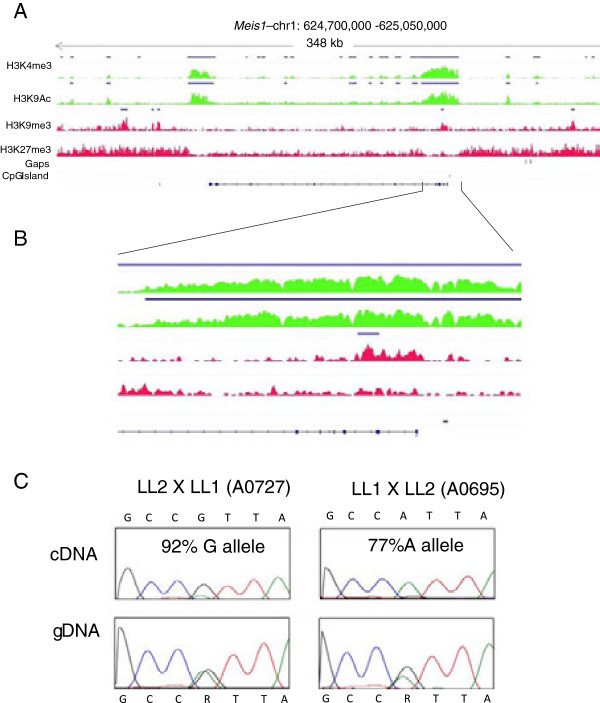
***Meis1 *is maternally imprinted in *M*. *domestica *fibroblasts. A)** Histone profiles of *Meis1*. Two MOAs (two green scans), two MORs (two red scans), assembly gaps, annotated promoter CpG island, and the gene annotation. Significant histone peaks (MACS, p ≤ 10^-5^) are indicated by blue bars above the scan peaks. **B)** Enlargement of the genomic segment including and flanking both sides of the promoter region of *Meis1*. **C)** Genomic DNA and cDNA genotypes of one animal from each reciprocal cross. Percent of maternal allele contribution as determined by pyrosequencing is shown in each box.

Among the 17 candidate loci described above, three annotated genes with promoters concurrently marked by the two MOAs and H3K9me3 (MOR) were clearly heterozygous for SNP variants in gDNA and showed strong allele-biased expression of alleles in cDNA: *Meis1* (ENSMODG00000003396), *Cstb* (ENSMODG00000021035), and *Rpl17* (ENSMODG00000011184) (Figure 3A-C; Additional file 1: Table S8, Additional file 2: Figure S2). *Meis1* showed unambiguous parent-of-origin-specific differential expression (Additional file 2: Figure S3). Quantification of relative maternal vs. paternal allele expression levels by pyrosequencing showed 92% and 77% expression of the maternal allele for one animal from each reciprocal cross (A0695 and A0727 respectively) (Figure 3C). Four additional F_1_ animals were examined for monoallelic expression, and informative animals showed strong maternal-allele biased expression with an average of 82% of transcripts originating from the maternal allele. Both *Cstb* and *Rpl17* also exhibited monoallelic expression; however, the pattern for *Cstb* was not parent-of-origin-specific, but showed allele-specific expression bias (Additional file 1: Table S8; Additional file 2: Figure S3). *Rpl17* allelic transmission direction could not be determined from the families examined, so distinguishing between imprinted vs. allele-biased (non-imprinted) expression was not possible for this locus. *Igf2r* also showed monoallelic expression in the one F_1_ animal (A0695) that was heterozygous for a trackable SNP (Additional file 2: Figure S3), but no informative reciprocal cross type was present in the families examined.

### Methylation states of promoters

**Figure 4:**
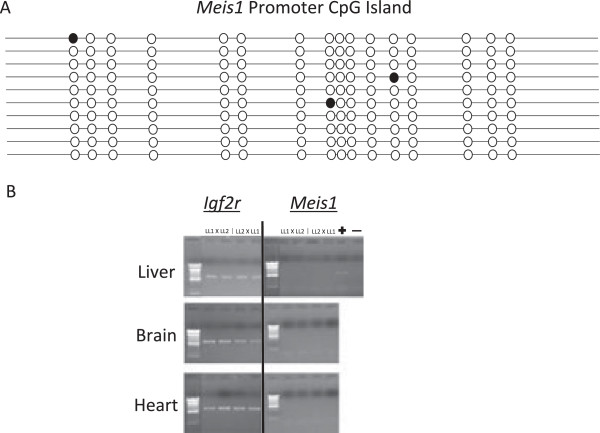
**DNA methylation profiles of *Meis1 *and tissue-specific expression pattern. A)** Bisulfite converted DNA was cloned and sequenced. Each line represents an individual PCR product. The annotated promoter CpG island was assayed for DNA methylation. Unfilled circles represent unmethylated cytosines at CpG dinucleotides and filled circles represent methylated cytosines. **B)** 1% agarose gel of PCR amplicons (cDNA) generated from liver, brain, and heart mRNA from four F_1_ animals used in this study (A0690. A0695, A0716, A0727). *Igf2R* is expressed in all three tissues, but *Meis1* is not expressed. Ladder = 100 bp DNA Ladder; Positive control = fibroblast cDNA.

We next utilized bisulfite sequencing to assay cytosine methylation at promoter CpG islands of the four monoallelically expressed genes (bisulfite PCR primers are listed in Additional file 1: Table S9). For *Meis1*, we assayed 16 CpG dinucleotides across the promoter and found a hypomethylated state but no evidence of differential methylation (Figure 4A). The promoters of *Cstb*, *Rpl17*, and *Igf2r* were also hypomethylated with no evidence of differential methylation. Recently, Das *et al*. (2012) discovered a differentially methylated CpG island in intron 11 of *Igf2r* in the liver, brain, and kidney of *M. domestica*. We assayed 18 CpG dinucleotides across this same CpG island and found this DMR in fibroblasts as well (Additional file 2: Figure S4). However, we were unable to assess allele-specific methylation patterns, as a parent-of-origin specific SNP was not present in this region in our animals. The hypomethylated states of the promoters of *Meis1*, *Cstb*, *Rpl17*, and *Igf2r*, as well as the DMR in intron 11 of *Igf2r*, were also verified in three other F_1_ animals: A0690 (female), A0716 (male), and A0727 (male).

## Discussion

Of the 35,105 putative promoters assayed in our ChIP-seq analysis of *M. domestica* fibroblasts, only ~46% (16,320) were marked by H3K4me3. This fraction is considerably smaller than the 74% and 71% of promoters marked by this expression-associated modification in cultured human and mouse cells, respectively [29,45], and is most likely an artifact of inaccuracy in the annotation of the *M. domestica* gene set. The initial set of predicted protein-coding and non-coding genes was produced by analyzing similarity with well-annotated eutherian gene sets, a practice that is expected to underrepresent or overlook diverged orthologs, paralogs, and marsupial-specific genes [46,47]. Further annotation has relied on individual sequencing of genes-of-interest, as well as a small number of RNA-seq data sets that are enriched for the 3’ ends of genes, leaving the 5’ annotation of many genes incomplete or inaccurate. This issue was underscored by a recent, comprehensive RNA-seq study of the *M. domestica* X chromosome [40] in which we found that the 5’ ends of nearly half of the genes on the X chromosome are incorrectly annotated in the MonDom5 assembly, with ~30% having a transcription start site more than 5 kb upstream from the first annotated 5’ exon. Annotation issues of this kind, especially at the 5’ ends of genes, pose a significant challenge for correlating promoter histone modification states with transcriptional states. In light of this limitation, our results likely underestimate the number of opossum promoters marked, either independently or concurrently, by MOAs and/or H3K9me3.

We were, nevertheless, able to identify 179 genes that were concurrently marked by MOAs and H3K9me3 within 5 kb of an annotated 5’ exon. Twenty-one of these were expressed in fibroblasts and had an informative SNP in each reciprocal cross. Importantly, only six of them showed 100% overlap of significant peaks of H3K4me3, H3K9Ac, and H3K9me3, and half of these exhibited strongly biased allele expression, of which at least one, *Meis1*, was clearly expressed in a parent-of-origin specific manner; i.e., is imprinted. None of the 11 candidate genes with less than 100% peak overlap exhibited monoallelic or strongly skewed expression. The high frequency (50%) of monoallelic expression among genes with 100% overlap of transcriptionally opposing histone marks suggests that complete peak overlap be adopted as an essential criterion in future *ab initio* searches for imprinted genes in non-eutherian species.

It is important to note that opossum *Meis1* expression occurs *in vivo* as well as in cultured fibroblasts. For example, in a study unrelated to the present one (and unfortunately uninformative for imprinting analysis), *Meis1* transcripts were found to be abundant in RNA-seq reads in cDNA prepared from gestational day 13.5 (E13.5) opossum brain and extra-embryonic membranes (X Wang, KC Douglas, AG Clark, PB Samollow, unpublished data). In addition, expression of the *Meis* family of genes in eutherians is strongly developmental-stage and cell-type specific, and inspection of transcript contig assemblies in the genome browser features of OpossumBase (http://opossumbase.org/?q=genome_browsers) [48] indicate that expression of *Meis1* is also developmentally variable and tissue-specific in opossum. The OpossumBase contigs were constructed from normalized cDNA libraries and are not useful to gauge expression levels, but they do verify that *Meis1* transcripts were sufficiently abundant to construct full-length mRNA transcript contigs from E9.5 embryo and E12.5 fetus samples, and from 1-day and 12-day post-partum newborns, but not from 25-day newborns. Similarly, transcripts sufficient for full-length contig assembly were present in adult ear pinna, thyroid, eye, tongue, heart, pancreas, stomach, colon spleen, ovary, and skeletal muscle, but not in adipose, brain, lung, diaphragm muscle, liver, kidney, or testis samples. In the present study, using standard PCR protocols, we unable to detect *Meis1* 3'UTR transcripts in adult opossum liver, kidney, and heart in cDNA prepared from four F_1_ animals from our reciprocal crosses (Figure 4B). This outcome agrees with the contig profiles in OpossumBase for liver and kidney, but not for heart; which suggests that *Meis1* is expressed in heart, but at levels too low for detection by routine PCR amplification from non-normalized cDNA.

Of the two remaining monoallelically expressed genes, *Cstb* clearly showed allele-biased expression that was independent of parent of origin, while imprinted vs. allele-biased expression of *Rpl17* could not be distinguished due to lack of reciprocal allelic transmission data. The possibility of different underlying causes for monoallelic expression emphasizes the importance of conducting reciprocal crosses to detect genuine parent-of-origin-specific expression patterns, a practice that has been absent from many past studies of marsupial imprinted genes.

Assessment of the transcriptional state of these three monoallelically expressed genes reveals the first case of an imprinted gene in a marsupial that is not known to be imprinted in any other organism, and suggests a role for histone modification states in the occurrence of monoallelic-expression of genes in the opossum and perhaps other marsupial genomes. Contrastingly, methylation analysis of gDNA from these fibroblasts failed to find evidence of DMRs at annotated CpG islands in the promoter regions of this novel imprinted gene or either of the other monoallelically expressed genes, *Cstb* and *Rpl17*. This is consistent with past reports that DMRs are rare or absent from marsupial orthologs of eutherian imprinted genes.

Examination of the four previously known annotated opossum imprinted genes, *Igf2r*, *Htr2A*, *L3mbtl*, and *Mest* failed to detect transcriptionally opposing histone modifications at their respective promoters or their gene bodies. *Igf2r* is not imprinted in humans but is imprinted in mouse, sheep, dog, and marsupials (wallaby and opossums). In mouse, the transcriptional regulation of *Igf2r* is controlled by a DMR in intron 2 and by an antisense transcript (*Air*). Interestingly, the DMR at intron 2 is present in human, mouse, and sheep, but absent in dog and marsupials [49,50]. Transcriptionally opposing histone states have been associated with the imprinted state, or lack thereof, in human and mouse; but the full-length *Air* antisense transcript has only been described in mouse [51,52]. *Htr2A*, *L3mbtl*, and *Mest* show variation of imprinted status in human organs sampled, and are associated with certain disease states that correlate with aberrant DMRs, but no studies of associated histone states have been reported for these loci [53-55].

We were able to assess the imprinting status at the *Igf2r* locus, but a lack of suitable SNP variants in our animals prevented us from analyzing expression patterns of *Htr2A*, *L3mbtl*, and *Mest*. It is possible that these genes are not imprinted in opossum fibroblasts, in which case the absence of transcriptionally opposing histone modifications would be expected. Alternatively, any or all of these three genes could be imprinted in opossum fibroblasts but not marked or regulated by the specific histone modifications we examined, or DMRs, but rather by some yet-to-be-identified genomic elements or regulatory mechanisms such as non-coding RNA transcripts. If so, there could be additional imprinted loci in fibroblasts that went undetected by our strategy relying on only four histone modifications.

Although *Meis1* showed parent-of-origin-specific allele expression in three individual fibroblast cell lines, there was ‘leaky’ expression of the paternal allele in some samples. Leaky expression of the repressed allele has been observed for some imprinted genes in eutherians and for some paternally imprinted X-linked genes in marsupials [7,8,56-58]. At the *G6pd* locus, the degree of paternal allele leakiness is age-dependent, with adults showing greater levels of paternal leakage than fetuses and newborns [59]. Similarly, studies in eutherians have demonstrated a loss of allele-specific gene regulation for X-linked genes in a passage-number-dependent manner in primary cell lines [60]. Although we used low passage fibroblast cell lines, the cells were originally grown from adult tissue, and the combination of adult source and increasing passage could have resulted in higher levels of leakiness. Alternatively, it is possible that the epigenetic regulation of imprinted loci in marsupial cells is not as stable as in eutherians due to the apparent lack of differential DNA methylation at these loci. Furthermore, most studies of marsupial imprinted gene expression have not utilized highly sensitive assays, such as pyrosequencing, to measure allele-specific expression of imprinted genes; so leaky expression of the repressed allele could be more prevalent than previously believed.

The vertebrate *Meis* gene family comprises three homeobox genes, which act as cofactors for a wide range of Hox genes. Acting alone or in combination with other Hox cofactors, especially members of the *Pbx* transcription factor family, *Meis* family genes influence myriad early developmental processes that are essential for body axis patterning and organogenesis [61-66], neurologic (brain, eye) development [62,64,66-69], cardiac development and cariomyocyte regeneration [70,71], hematopoiesis and angiogenesis [61,69,71-74], and more in mouse, zebrafish, chicken, and *Drosophila*. In the absence of protein functional data, we are unable to determine experimentally which ortholog of the vertebrate *Meis* gene family is represented by the imprinted opossum *Meis* locus. Nevertheless, a reciprocal blast search strategy using the opossum predicted mRNA and amino acid sequences from the Ensembl annotation indicates that the opossum-imprinted *Meis* gene shares the greatest sequence similarity with *Meis1* orthologs of human, mouse, and rat. Moreover, this locus was matched by the Illumina reads to the exclusion of other *Meis* paralogs, and comparative synteny analysis shows gene content and order of the genomic region flanking this locus to be strongly conserved with that containing the human *MEIS1* and mouse *Meis1* genes. Hence we feel confident that this is the ortholog of *MEIS1*/*Meis1*.

Although *Meis1* expression is crucial in many fundamental embryonic and fetal developmental processes, how its functions relate to current views on the evolutionary advantages of genomic imprinting is not obvious. The highly developed and widely accepted Parental Conflict Model (a.k.a. Kinship Model) proposes that imprinting will be favored when the extraction of resources from a parent by its offspring occurs in such a manner that the fitness benefits of provisioning the offspring differ between the two parents [75-78]. Applied primarily to therian mammals because of their universal placental/lactational provisioning strategy (both eutherians and marsupials form placental attachments), the Conflict Model juxtaposes the reproductive strategies of males and females by noting that offspring of different fathers in multiple-paternity litters compete for the same maternal resources. Maximization of fitness for any one father is achieved by his progeny extracting maternal resources more effectively than the progeny of other fathers, whereas for the mother, the best strategy is to provide resources equitably among all her offspring. This creates "conflict" between paternal and maternal genomes for genes that influence resource allocation and is generally couched in terms of fetal growth regulation, and/or in neurologic development that can enhance or inhibit postnatal growth rates through variation in feeding competence [75,77-79]. The Conflict Model is pleasingly consistent with the known roles of several imprinted genes in fetal growth and postnatal nutritionally related behaviors, but for most imprinted genes agreement with the Conflict Model has been assumed rather than demonstrated [12].

The known influences of *Meis1* in vertebrate (and *Drosophila*) development are ambiguous with regard to the Conflict Model. Proper *Meis1* expression is essential for body and limb bud axis patterning, brain segmentation, angiogenic patterning, and the proliferation of stem cells in developing organ systems such as retina, heart, and hematopoietic centers. However, *Meis1* expression also has inhibitory effects that induce cell-cycle arrest and stem cell quiescence in ways that enable non-proliferative cellular differentiation during and after organogenesis while simultaneously preserving pools of multipotent stem cells for lineage renewal [61-64,66,68-74]. Experimental alterations of *Meis1* expression are associated with severe, often fatal, prenatal developmental defects in neurologic patterning, the differentiation of hematopoietic stem cells and establishment of definitive hematopoiesis, peripheral angiogenesis, cardiomyocyte cell-cycle regulation, and hematopoietic cancers; but in no case has *Meis1* expression been associated with fetal or postnatal growth rates or nutrient acquisition *per se*. Together with the absence of *Meis1* imprinting in human and mouse, the conflicting developmental functions of this gene suggest no obvious reason why it should be imprinted in opossum. Perhaps the explanation for *Meis1* imprinting in opossum needs to be sought outside of the confines of the Conflict Model.

In view the logical power and broad acceptance of the Conflict Model, few alternative hypotheses for the advantages of imprinted gene expression have been proposed, and the few that have were quickly dismissed as evolutionarily unstable or logically flawed. Nevertheless, as there is no *a priori* basis for believing that the Conflict Model must explain every case of imprinting in all species, there could be as-yet-unidentified biological advantages for the imprinting of genes that are not involved in embryonic and perinatal growth and development. It seems prudent, therefore, to remain open to, and actively seek, alternative hypotheses for the evolutionary advantages of imprinting on a locus-by-locus basis, especially in non-eutherian species.

## Conclusion

In this first comprehensive report on histone profile states in any marsupial species, we have described the genomic landscapes for four canonical histone modifications, H3K4me3, H3K9Ac, H3K9me3, and H3K27me3 and successfully identified a novel imprinted gene in opossum as well as two other monoallelically expressed genes. These results demonstrate the practicality of an *ab initio* strategy for discovering imprinted genes in non-eutherian mammals and, potentially, non-mammalian species as well. Overall, the findings support the conclusion that specific histone modifications are conserved features that mark the promoters of some imprinted genes in all therians, but also suggest that marsupials use multiple epigenetic mechanisms for imprinting, some of which are distinct from those known in eutherians; e.g., DNA methylation appears to play little, if any, role in regulating the imprinted state in marsupial mammals. Furthermore, while the imprinting status of some genes is conserved across therians, identification of a marsupial-specific imprinted locus, *Meis1*, which is not known to be imprinted in any eutherian species examined, bolsters the concept that lineage-specific differences in selective pressures may have led to phylogenetically distinct variants of the imprinting phenomenon.

## Methods

### Animals and tissue collection

For the ChIP-seq experiments, animals from two laboratory stocks (LL1 and LL2) of the opossum, *M. domestica* were utilized [80]. For initial ChIP-seq profiling, primary fibroblasts were cultured from ear pinna of a male F_1_ (ID# A0514) from an LL1 X LL2 mating and collected using standard methods (Additional file 2: Figure S1A). For further experiments, reciprocal crosses were conducted between LL1 and LL2 stocks, and primary fibroblast lines were established from ear pinnae collected from the parents in each cross, and from four F_1_ (LL1 X LL2) and four F_1_ (LL2 X LL1) individuals using standard methods (Additional file 2: Figure S1B). All procedures involving opossums were approved by the Texas A&M University, College Station, Institutional Animal Care and Use Committee (TAMU Animal Use Protocols 2011–141 and 2011–191).

### Chromatin immunoprecipitation (ChIP) and ChIP-Seq

Native-ChIP (N-ChIP) was conducted on low (< 5) passage, primary fibroblasts from male A0514 using a method modified from Dindot *et al*. [28]. Harvested fibroblast cells were washed in PBS and homogenized in 500 μL of Buffer I (0.3 M sucrose, 60 mM KCl, 15 mM NaCl, 5 mM MgCl_2_, 0.1 mM EDTA, 15 mM Tris, 0.5 mM DTT, 0.1 mM PMSF). The sample was centrifuged for 5 min. at 3000 × g, the supernatant was removed, and the pellet was re-suspended in 200 μL of Buffer I. Cells were lysed on ice for 5 minutes by adding 200 μL of Buffer II (Buffer I + 4 μL of NP40), and nuclei were isolated by centrifugation for 20 minutes at 10,000 × g through 1.5 mL of Buffer III (1.2 M sucrose, 60 mM KCl, 15 mM NaCl, 5 mM MgCl_2_, 0.1 mM EDTA, 15 mM Tris, 0.5 mM DTT, 0.1 mM PMSF). The nuclei-enriched pellet was washed with Buffer I, centrifuged, and re-suspended in 350 μL of micrococcal nuclease digestion buffer (0.32 M sucrose, 4 mM MgCl_2_, 50 mM Tris, 0.1 mM PMSF). Chromatin was digested using 10 units of micrococcal nuclease (Sigma, N5386) for 10 minutes at 37°C. The reaction was stopped using 50 μL of 0.5 M EDTA.

For an input control, 100 μL of digested chromatin was removed before treatment with antibodies and the DNA fraction was extracted. For ChIP, 4.0 μg of digested chromatin was incubated at 4°C overnight with one of the following antibodies: anti-H3K4me3 (Millipore #07-473), anti-H3K9Ac (Millipore #CS200583), anti-H3K9me3 (Millipore #07-442), anti-H3K27me3 (Millipore #07-449), or non-specific, rabbit IgG (Millipore #12-370). Antibody-bound chromatin was isolated using Dynabeads® Protein A (Invitrogen), washed, and eluted according to manufacturer’s specifications. N-ChIP and input DNA were purified using Qiagen MiniElute Spin Columns (Qiagen, CA) and enrichment was verified using real-time PCR (data not shown). Non-indexed Illumina libraries were constructed at Global Biologics, LLC (Columbia, MO) and sequenced on an Illumina GAIIx at the University of Missouri–Columbia DNA Core Facility using 51-or 101-base chemistry. Image analysis and base calling were performed using Illumina software.

### ChIP-Seq analysis

Raw sequence reads were filtered for quality and mapped to the MonDom5 genome assembly using Bowtie in the Galaxy suite [81-83]. A seed length of 28 bases was used with a maximum of 2 mismatches permitted between the seed and reference genome, and only the best alignment reported for each read. Significant peaks of enrichment were identified for each histone modification using Model-based Analysis for ChIP-seq (MACS) using the input control option [84]. The ChIP-seq data were deposited in the GEO database under accession number GSE47723. Ensembl gene models (release 64) were used and annotated CpG island coordinates were obtained from the UCSC genome browser [85]. Putative promoters were defined as regions 5,000 bases upstream to 500 bases downstream of annotated transcription start sites. To provide a co-occurrence criterion for inclusion of genes in the imprinted-candidate pool, genomic features (promoters, CpG islands, histone modification peaks) were considered overlapping if they shared one or more bases in common. Overlaps between features were assessed using scripts in the BEDTools package [86]. In order to be considered a candidate-imprinted gene, the putative promoter of the gene had to be concurrently marked by significant H3K4me3, H3K9Ac, and H3K9me3 peaks, and contain an annotated CpG island.

### SNP discovery in candidate-imprinted genes

PCR primers were designed using Primer3 [87] to amplify 600–700 bases of the putative 3’-untranslated region (UTR) of each candidate-imprinted gene as well as *Igf2r* (Additional file 1: Table S3). Genomic DNA (gDNA) was extracted from livers of the eight individuals comprising the P generations of each cross using standard protocols and was PCR amplified (20 μL reaction volume) for each primer set using AmpliTaq Gold polymerase (Invitrogen). After an initial denaturation of 5 minutes at 95°C, 38 PCR cycles were conducted at 95°C for 30 seconds, 54°C for 30 seconds, 72°C for 30 seconds, followed by a final extension for 7 minutes at 72°C. PCR optimization was conducted where necessary. To confirm PCR amplification, 3 μL of PCR product was run and visualized on a 1% agarose gel (data not shown). All PCR products for each of the eight parents were pooled, eight indexed Illumina libraries were created from each pool, and 101 bases were sequenced on an Illumina GAIIx at the University of Missouri–Columbia DNA Core Facility. Raw reads were filtered for quality, mapped to the MonDom5 genome assembly, and SNPs variants were called using MPileup in the SAMTools package [88]. Variant regions were required to have a minimum of 20× coverage to be considered as candidate SNPs.

### Verification of imprinting status

Total RNA and gDNA were extracted from six of the eight fibroblast cell lines from the F_1_ generation using standard protocols (two F_1_ animals, A0703 and A0716, are absent from the SNP analysis due to the lack of success in establishing fibroblast lines from these animals). Total RNA was treated with DNase I and converted to cDNA using the SMARTer cDNA Synthesis Kit (Clontech). PCR reactions were conducted as previously described, and gDNA and cDNA PCR products were sequenced on an ABI 3730XL at Beckman-Coulter Genomics, Inc. (Danvers, MA). Sequences were viewed in Sequencher4.10™.

To quantify maternal/paternal allele expression ratios, pyrosequencing PCR was conducted on cDNA from one F_1_ male and one F_1_ female from each of the LL1 X LL2 and LL2 X LL1 crosses. Pyrosequencing PCR and sequencing primers were designed using the PyroMark Assay Design Software Version 2.0.1.15 (Qiagen, CA). Pyrosequencing PCR amplification was carried out in a 40 μL system using Ampli-Taq Gold polymerase under the following cycling conditions: 1 cycle of 95°C for 5 min; 45 cycles of 95°C for 45 sec, 57°C for 30 sec, and 72°C for 20 sec; followed by 1 cycle of 72°C for 10 min. PCR products were prepared according to the manufacturer’s protocol and loaded on the PSQ 96MA Pyrosequencer with PyroMark Gold Reagents (Qiagen, CA) using the Allele Quantification method (AQ). Two technical replicates were done for each gene in each sample. Overall, variation between replicates was negligible (≤3%), and the final expression percentages were determined by averaging the results from each run.

### Analysis of CpG island methylation

To assess the methylation status of promoter CpG islands, gDNA was isolated from fibroblasts from two F_1_ animals from each reciprocal cross (4 total animals) and treated with sodium bisulfite to convert unmethlyated cytosines to uracils using the Qiagen EpiTect Bisulfite Kit (Qiagen, Inc). PCR primers were designed to amplify bisulfite converted DNA using Methyl Primer Express Software (Applied Biosystems, Inc). BS-PCR products were gel purified, sub-cloned using the TOPO TA Cloning Kit (Invitrogen), and blue/white screened using XGal (40 mg/mL). For each cloned PCR product, plasmids were purified from at least 16 positive white colonies and were sequenced at Beckman Coulter Genomics by the Sanger dideoxy-chain termination method using the M13 forward primer. Sequences were inspected and analyzed using Sequencher4.10™.

### Availability of data

Raw Illumina sequence files and MACS peaks can be found at the Gene Expression Omnibus (GEO) under accession no. GSE47723.

## Abbreviations

3'-UTR: 3'-Untranslated region; ChIP-seq: Chromatin immunoprecipitation followed by next-generation sequencing; DMR: Differentially methylated region; cDNA: Complimentary DNA; gDNA: Genomic DNA; ICR: Imprinting control region; LINE: Long interspersed nuclear element; MACS: Model-based analysis for chip-seq; MOA: Mark of activation; MOR: Mark of repression; SINE: Short interspersed nuclear element; SNP: Single nucleotide polymorphism.

## Competing interests

The authors declare that they have no competing interests.

## Authors’ contributions

KCD and PBS conceptualized the project. JLV developed the LL1 and LL2 animal stocks. KCD, MJ, and AW carried out the experiments. XW and AGC provided the pyrosequencing data. KCD conducted the data analyses, and KCD and PBS wrote the manuscript. All authors read and approved the final manuscript.

## Supplementary Material

Additional file 1 Supplemental Tables. Click here for file

Additional file 2 Supplemental Figures. Click here for file
