# Supplementary material for: Genome-wide histone state profiling of fibroblasts from the opossum, Monodelphis domestica, identifies the first marsupial-specific imprinted gene
Source: BMC Genomics. 2014 Jan 31;15:89. doi: 10.1186/1471-2164-15-89 (PMC3912494; doi:10.1186/1471-2164-15-89)
Supplement: Additional file 1 — Supplemental Tables. [file 1471-2164-15-89-S1.pdf]

Additional File 1

Supplemental Tables

Supplemental Table S1. Summary of ChIP-Seq data.

Histone Modification	Raw Reads ($X10^6$)	Filtered and Aligned Reads ($X10^6$)	Enrichment Peaks ($p \leq 10^{-5}$)	Overlap with Putative Ensembl Gene Promoters[2]
H3K4me3	97.0	71.3	79,412	16320
H3K9Ac	143.9	112.4	56,719	13420
H3K9me3	74.7	56.9	52,511 (159,734)[1]	4514
H3K27me3	143.2	118.0	16, 592	NA
Input	120.2	78.2	NA	NA

[1] significant peaks determined using MACs ($p \leq 10\text{-}3$)
[2] 500 bp upstream to 500 bp downstream of first annotated

Supplemental Table S2. Summary overlaps of significant peaks with each other, annotated CpG islands, and annotated putative promoters.

	H3K4me3	H3K9Ac	H3K9me3	K4me3+K9Ac	K4me3+ K9Ac+K9me3
H3K4me3 (n=79,412)	----	45,331	----	----	----
H3K9Ac (n=52,511)	47,275	----	----	----	----
H3K9me3 (n=56,719)	6,410	1,615	----	1,531	----
Putative Promoters (n=35,301)	16,320	10,959	3,163	13,176	253
CpG Islands (n=22,441)	11,580	9,061	188	9,319	240
Promoters + CpG Islands[1] (n=10,814)	7,871	6,759	773	6,803	136 (178)[2]

[1] Annotated CpG Islands within the 5.5 kb range of putative annotated promoters

[2] Number in parenthesis represents overlapping peaks at promoters and CpGs using a lower level of significance for H3K9me3 peak calls ($p \leq 10-3$)

Supplemental Table S3. Candidate-imprinted genes Ensembl Gene ID, Associated common gene name from Refseq annotation (if applicable), 3' UTR coordinates as annotated, and Forward and Reverse PCR Primers.

Ensembl Gene ID	Gene Name	Chr	Start	End	Identifier	Forward Primer	Reverse Primer
ENSMODG00000000633	VCL	chr1	50888333	50889333	1	GAACCTCATGCAGTCTGTGAAG	TGTTTGGTAAGGGAAAGCCTAC
ENSMODG00000006639	39145	chr1	78887646	78888646	2	TTGAACAGCCAGAACTCAAGAA	GGTTTACTTCAAACAGCAGCAA
ENSMODG00000008434	ATE1	chr1	86454736	86455736	3	ACGACAGAAAGGACCAAGTGAT	TATAGTTGCCCAAAAACCCATC
ENSMODG00000010523	DUSP5	chr1	101428590	101429590	4	TCCAGAGAAAGGTTCCAGTGTT	GGTTTGAAGGCTCTTGCTACAC
ENSMODG00000011165	CNNM2	chr1	108942899	108943899	5	CTGAACCTCTCCCGCATAATAG	TGAACTGACTCTTACGGAGCTG
ENSMODG00000019402		chr1	117607423	117608423	6	AGCTATGCTTGGGAGATCAAAG	AAGTTCCAGAACCAGTTTCCAA
ENSMODG00000011135	TPM1	chr1	148353086	148354086	7	CACATGCCAAAGAAGAAAACCT	CCCCCAACACAAATAAGAAACA
ENSMODG00000000464	C15orf23	chr1	197125347	197126347	8	TCCGACTTTGTAGAACCCAGAT	TTCATTCCAAGCCCACTATTCT
ENSMODG00000000164	EXD1	chr1	198202335	198203335	9	CCTTCAAGAACAGGGATAAATG	GGGGAAAGAAGATGATTGAGAA
ENSMODG00000000158	XM_001370957.1	chr1	198271412	198272412	10	GATTCAGGGTTCCTCTCCTCTT	GTAAGAACCATCCAGCCATCTC
ENSMODG00000003366	XM_001372294.1	chr1	228646013	228647013	11	ACTACAGGTCAAAAGGGCAGTC	GCCTGGATGTGAAAGTTATCAA
ENSMODG00000011362	SRSF5	chr1	275456081	275457081	12	TTTGATAAGCCTTCTCCTGCTC	CCTCATCTCACCACACTCTTTG
ENSMODG00000011684	GCH1	chr1	276674516	276675516	13	CCCAGCAATTGAATAAAGGAAC	CTACCCTTCCCCATAAACCATT
ENSMODG00000012905	PPP2R3C	chr1	285526641	285527641	14	ACCCCAGTCATCTCTTGTTTGT	CTGGAGGACACATGATTTTTCA
ENSMODG00000012822	KIAA0391	chr1	285630813	285631813	15	GAACATGGCCTACTGCTTCTTT	GCTTTGCCTTTATGTTCCTAGC
ENSMODG00000010724	XM_001377829.1	chr1	333227663	333228663	16	CATGAGGTTTCTCTCACCACTG	CCCCCATAGGAAGATTACCAA
ENSMODG00000023761	XM_001377862.1	chr1	333244982	333245982	17	TTCTCTCACCATGGACTCTCAA	TCATCCATTCCCAGAGTCAATA
ENSMODG00000010717	PCDHGC5	chr1	333338010	333339010	18	AGGAAAAGAAGTAGGGTCAGCA	TGAGGGGTGGAGAAGAATAAGA
ENSMODG00000009937		chr1	342089584	342090584	19	TGCAGTTGCACTACTAGGCATT	CAGACAAGTGGTGGAGAAATTG
ENSMODG00000009436	HNRNPF	chr1	343914253	343915253	20	CATGTCCAGTATGAGTGGTGGA	ACCACTTTAACAGGGAATGGTG
ENSMODG00000007182	PANK3	chr1	367060313	367061313	21	TTGCACCAACTAATGGAAAGTG	TACAGGGATACTTCGGAGCAGT
ENSMODG00000006223		chr1	372593415	372594415	22	ATACAACCAACCTTGGCAACTC	AACCTCCACAACCACAGTCTTT
ENSMODG00000003806	SQSTM1	chr1	385459213	385460213	23	GGGGAATGCTTAAGATTTGTGT	CACCCCATCCCTAAATGAGAT
ENSMODG00000001669	SPAG4	chr1	389035953	389036953	24	AGAAATGGGGCATCTGAAACTA	CACACGTCTGATGAAGGTTTTT
ENSMODG00000019490	BCL2L1	chr1	401848708	401849708	25	ACAGGACACCTTTGTGGAACTT	GGCATGGTTTGGAGTTACTAGG
ENSMODG00000019496	ID1	chr1	401900281	401901281	26	TCCACACATAGATGGCTGTCTT	TGAACTCACCACTTCCCTCTCT
ENSMODG00000012957	LONP2	chr1	436892842	436893842	27	AAACCAGGCTTGGTCTCATAAA	AAGTAGGGTGAGCCAAATCCTT
ENSMODG00000017051	PRPF6	chr1	472620853	472621853	28	TTCAGTGTCACCTGCAATTTCT	AGGAATGTCCCAACTCAGGTAA
ENSMODG00000016314	CEBPB	chr1	495758719	495759719	29	ATCCCCTTTCTTCACTCGAATA	AAAAGAAGGTGGAGCAGTTGTC
ENSMODG00000016229	TP53RK	chr1	499725383	499726383	30	ACTAAGAGGAAGGAAGCGGTCT	GCCTTTAATGGAAGGAGGATTC
ENSMODG00000015575	FOSL2	chr1	510096298	510097298	31	CTTGAACTCTCCCACTCTCCTG	GAGCTATGCCTCTCATCTCCTG
ENSMODG00000014618	ID2	chr1	531430233	531431233	32	AGGAAGCCACAGTTTGAACATT	TTCTGAATTCCCCTCAGAGCTA
ENSMODG00000011515	TIA1	chr1	563790769	563791769	33	TGGGTGCAAATTATGGAGTACA	TGCAATTATCGATCCATCTGAG

ENSMODG00000011328	HK2	chr1	564790600	564791600	34	GCCCTTTTTCCTTTTACAGCTT	ACTCCCATGTCTGGAAGTCAGT
ENSMODG00000008637	ELP3	chr1	589524319	589525319	35	AGAAGGCAATGCATACAACTCC	TCTGAGCAGGAGACTTCCTTTC
ENSMODG00000006163	XRN2	chr1	600377628	600378197	36	GTGGTCCAGAAATTCTCAGACAG	GATATGGCCCTGGAAGGAATTA
ENSMODG00000005428	DSTN	chr1	605428140	605429140	37	TTTCACGATCACTCCAGAATTG	TATTGCCGAGAAACTAGGAGGA
ENSMODG00000003919	FERMT1	chr1	621177657	621178657	38	CCAGGTTACCATAGAGTTTGACC	GTGGCCCAAACATCAAGTTAGA
ENSMODG00000003396	MEIS1	chr1	624802971	624803971	39	GAATTGTTTAGGGCGGGTTTAT	TCATGCTGACTCTACTGCTTGA
ENSMODG00000002285	AHSA2	chr1	631466134	631467134	40	AAATTGGGTCAAGGGAACTAGG	GTAGCTTTTGGCAATCCTGAAC
ENSMODG00000001803	RTN4	chr1	639694124	639695124	41	AAGCAAAGCGGAGTAAAATCAG	CATCCCAAGTGTGTGATGAAAT
ENSMODG00000008750	MORN2	chr1	662002860	662003860	42	TTCGTGCCTACATTGTGAGTTT	ATGAATGGAATTGGAAGACTCG
ENSMODG00000008486	CYP1B1	chr1	663121133	663122133	43	TGAGCTATGGCAGAATTTGGTA	ATGTGCATCACTTTCAGCTACA
ENSMODG00000003201	HAS3	chr1	698983517	698984517	44	TTATTGCCAGGACCTTTTCAGT	TGCTACCGGAAACTTTTAATCC
ENSMODG00000011200	NAGK	chr1	704999526	705000526	45	AGCTCGTCTTACAGCAGAGGAA	AGAAGATCAACGGCTTTTTCAC
ENSMODG00000009450	KDM3A	chr1	716933050	716934050	46	GGTGGTTTCTACCACACCAAGT	ATACTCAAAGCCAACGAGTGTG
ENSMODG00000009386	IMMT	chr1	717367383	717368383	47	CCTCCTACAGCATCGAGCAC	GGGTGGATTCTTTCGTGATTTA
ENSMODG00000015604	MAT2A	chr1	717916461	171917461	48	AAGTTAAAAACCCTGGGAGGAG	AGTTGGGCTCTGCTATTCTGTC
ENSMODG00000001321	ALDH9A1	chr2	57215440	57216440	49	GGAGGAACAGACAGAACAATCC	ACTTCCTCTTTCCCCATGATGT
ENSMODG00000007194	XM_001366600.1	chr2	78706632	78707632	50	AGCATTTATTCAGGTGCTGTCC	TTAGTCCAGCAAGAAGTGAGCA
ENSMODG00000011721	PTGS2	chr2	82226320	82227320	51	AGGAAATGTCCATTAGCCAGAA	TGCATTCAGTGTTCAAGACTCC
ENSMODG00000001558	PPP1R15B	chr2	109874517	109875517	52	GGATTTGCATTGCAGACTCTAA	TGACATTTGAGCACAGACAGAA
ENSMODG00000004622	MARK1	chr2	131134701	131135701	53	GTTCAGTGGGAGATGGAAGTTT	TATCAATTAGCCCAAGGCAAAG
ENSMODG00000006246	CDC42BPA	chr2	139634610	139635610	54	ATCTGTGCTCAACAGCATCTTC	AACTGCTTCAGCATTCAGATCA
ENSMODG00000008286	B3GALNT2	chr2	154429949	154430949	55	GGGTAAGTTTACAGGCCAAATG	CCAGTCCGAACATAAACTCTCC
ENSMODG00000014128		chr2	169499702	169500702	56	TGCATGTTGGGTGTATCCTAAA	CAGCATGGATGAAGACTTTGAG
ENSMODG00000016946	LMNA	chr2	191002496	191003496	57	CAGCATCATGTAACCTGGAGAA	GAGCAGAGACTCAAACTCAGCA
ENSMODG00000014859	PTRF	chr2	194012782	194013782	58	AGAAGATTCGAGAGGGAGAGGT	CTACTGCTCCAAATCCCAAACT
ENSMODG00000014651	EIF1	chr2	194867160	194868160	59	TAAAACCAGTTTGGGAGGAATG	TGCTCAGCCTGAAACTCAAATA
ENSMODG00000024021	RPRML	chr2	206114036	206115036	60	GCAACCTGCTTATCAAGTCTGA	CCGAGAGACAGAGGGAGAGATA
ENSMODG00000007640		chr2	213236964	213237964	61	AATAGCAGGGGAGAAGGGTAAC	ACTCCCCACAGGTGAATGTAAA
ENSMODG00000004465	FAM20A	chr2	225162279	225163279	62	ACTCTGACCAAGCAGGTTTAGC	CTACCTCACAGGGTTGTTGTGA
ENSMODG00000003779	CEP95	chr2	229916066	229917066	63	GAAAATTCTTCAGCCTGGAACT	GACGATGACCTCTTCTTTCGAG
ENSMODG00000003743	DDX5	chr2	229961937	229962937	64	GCATGGCCTTCCAATAAAGATA	TAAAAATGCAGCCCTTCTATGG
ENSMODG00000003701	POLG2	chr2	229995182	229996182	65	ACAATGAAGGAGCTAATGCACA	CCACCATAAATTCCATTCTGAC
ENSMODG00000007792	VAMP2	chr2	254268634	254269634	66	AGGGCTGATGAAACTCCAGATA	TGTCGTGCAAATAAGGGTAGAA
ENSMODG00000008266		chr2	260064845	260065845	67	TCTTCTGGATAGAAGTGGCAAT	CAATCAACATACATGGGAGAGG
ENSMODG00000013704	MAPK14	chr2	275269390	275270390	68	GTTCTTTCCCTTCCTTCTAGGC	CCCCCAAGACATTCTTCTCTCT
ENSMODG00000012785	XM_001370586.1	chr2	285218809	285219809	69	TAATCCTTATCTCCGGCCTGTA	GGCTTTGTAGGTCAGAGGAAGA
ENSMODG00000018800	GCLC	chr2	302215607	302216607	70	AGGTGCTGGAAAGCAAAGTTAT	GATGGATGAGGGAGTTTGTTTC
ENSMODG00000018721	BAG2	chr2	307679005	307680005	71	AATTGGATGTGCTCTCGAAGAT	ACTTTTCTATGGCCAGGGATG

ENSMODG00000018444	IRAK1BP1	chr2	332904143	332905143	72	AAAGAATGGGAAGGGCAGATAG	TGTAGTTTGATTCCCCAACTGT
ENSMODG00000006175	XR_030477.1	chr2	451158631	451159631	73	CAAAAAGCTTGAAGACCTCACA	CTGACACGGACTCATCAACAGT
ENSMODG00000004472	F3	chr2	459548415	459549415	74	CACCGGTTTCAAAGTGTCTGTA	TTTTGTCATCATCATCCTCGTC
ENSMODG00000004326	CNN3	chr2	460038121	460039121	75	ACCTAAAACTTCTGCAGGCTCA	AATACATAACGGAAGCCAAGGA
ENSMODG00000005319	TBX15	chr2	491245071	491246071	76	GTTTCAGGAGGTCTGGTTCACT	ACTCTGTTCATCTCCCTCCAAC
ENSMODG00000018801	MCL1	chr2	495754229	495755229	77	GTTCTAAATCACCTGGGCAAAG	GTAGGAGCTGGTTTGGCATATC
ENSMODG00000019221	WSB1	chr2	505025234	505026234	78	CTCGTCGAGTTTCTTTGCTACC	AAACAATATGGCCGTGAGAAAC
ENSMODG00000000570	XM_001362687.1	chr2	520269915	520270915	79	TCCTACCCATGAGGGATTTTTA	GGGTCTTCACAAAGATCTGCAT
ENSMODG00000016394	UBC	chr2	520272577	520273577	80	GAGCCCAGTGACACTATTGAGA	GTCCTGCCACTGCAAGATTTAT
ENSMODG00000023140		chr2	522386355	522387355	81	GAATGTGGAAAAGGATTTGGAC	CCCATAACTTTCTCCATTCCAG
ENSMODG00000019470	SNX18	chr3	16801021	16802021	82	TAGAGGGAGTTTTCCTCACCTG	GCAAAGACCCTGTCGTTTTAAT
ENSMODG00000025244	ENC1	chr3	49543210	49543819	83	CTGGTACCGTCCACCTGTTCT	GAGGACAGACCTTCATGTGTGA
ENSMODG00000015548	RIOK2	chr3	52330287	52331287	84	ACCATGCTCCAGCATAAGATTT	TTTCAGGAACTGGTGAAACAGA
ENSMODG00000003564	LIPG	chr3	69451400	69452400	85	CAACTACCCCTGCTACATAGGC	TCAGACCTCAGAATGGTGAAAA
ENSMODG00000000399	RPRD1A	chr3	87470085	87471085	86	ATCTTCCATTTGCTGGAGACAT	GACACAGTGAGTTGGGGTGATA
ENSMODG00000001446	IRX2	chr3	94853941	94854941	87	TGTGGTTCATCTACCAAAAAGC	GTCCACAGGTAGGTGTCACAAT
ENSMODG00000006950	JPH1	chr3	162249049	162250049	88	TTCAGTCATGATTGTCCTGGTC	CACTTCCACAGCAACAGTATCC
ENSMODG00000006961	TMEM70	chr3	162533985	162534985	89	TAAGCCAGGATCAGAACAGAGC	CCATCTTATGGGCTATGACAAA
ENSMODG00000010525	CEBPD	chr3	194975067	194976067	90	AAGCTGGTGGAGCTCTCTTCT	CAAGTTTCTCCGGAGTATGTCC
ENSMODG00000020132	NR2F1	chr3	208749463	208750463	91	TCGACAACATATATCGCACTCA	AGGTAAAACCCCCATTGAAACT
ENSMODG00000020286	FBXO4	chr3	232703757	232704757	92	GTTTGGTACACCACAAGCTTTC	TTTTCCTAGGTTCAGGATGTGG
ENSMODG00000020357	C5orf33	chr3	240202567	240203567	93	GATGATGAACTTCGAACTGTGC	AGCAGCATGATTTTCTCCAGAT
ENSMODG00000021462	TGIF1	chr3	269085279	269086279	94	ATCTGGACCAAGTGCAAATACC	CCCGGTCAATGCTAATAACCTA
ENSMODG00000006222	PABPC1	chr3	365999412	366000412	95	TCTACCAAACTGGCTTTTGACA	GCTGAGCAAGGAAACGTAATTT
ENSMODG00000004746	SYBU	chr3	377688004	377689004	96	CCTCCTAAGTGGTTCAGGAAGA	TCAAACCTAGAAGGTTGCCATC
ENSMODG00000001735	MYC	chr3	404001553	404002553	97	AGTGAGCTGCTGAGGAAAGAAC	TCAAGGAAATCCTTTCAAGCTC
ENSMODG00000000768		chr3	421671148	421672148	98	CCCTTAAGGATGGTTTTCCTCT	TGCTGAGGAGTTGGTATGAGAA
ENSMODG00000000764	KCNK9	chr3	421755076	421756076	99	CTCACTGCACATGCTTCATTTT	CTGGCCTACACAGCTTTACTGA
ENSMODG00000006967	JUNB	chr3	429190670	429191670	100	TGTAATTAAGACGCCAGGGAGTA	GACTCGAGGACAAGGTGAAGAC
ENSMODG00000011184	XR_030226.1	chr3	437574717	437575717	101	ACCAGAAATGGGAAAAGCTGTA	CAAAAACTTATGGCATGGGAGT
ENSMODG00000011203	PYCRL	chr3	437738813	437739813	102	CAAGAGCTGACCAAGCAGTAGA	AATGGAAGAGTCAGGACCTCAA
ENSMODG00000000718	DAPK3	chr3	440376529	440377529	103	GCTACAAGTAGCCTGTGCCTCT	AAGTGGCATCAGAGATGAGGTT
ENSMODG00000001178		chr3	443006006	443007006	104	GCCAAATTGGATCAAGTACAGG	CTCACTTCACCCGCAGATATTA
ENSMODG00000001236		chr3	443062526	443063526	105	GAGAAAGTGATGGAAAGAGAGAGA	GGGGATTTCAAGTCCTTTCACT
ENSMODG00000003841	XM_001376300.1	chr3	460433663	460434663	106	ACAAAGGAAGAACTGGCACTTG	GGACAGAAATCCAACAAACTCC
ENSMODG00000003520		chr3	475399984	475400984	107	GACCATTACCCTTGAAGTGGAG	CTTACCGGCAAAGATCAGTCTC
ENSMODG00000014519	ILVBL	chr3	477439465	477440465	108	AGAACAAAGGGACAAGCAACAT	CAGTTGTTGCTCAACTCTGACC
ENSMODG00000025637		chr3	499708709	499709709	109	GGAGAAGGTTCTGGGCTAATCT	GACCTTTTGAATGCAGTGAATG

ENSMODG00000023660		chr3	501254997	501255997	110	CAGAGATGTGACCTTTGGCATA	GGGAGAAACCTTATGAATGCAA
ENSMODG00000009557	MORC2	chr3	520340337	520341337	111	ACACGTGTACAGAGGCAGCAC	CCACTTTAGGACATCGACATCA
ENSMODG00000021035	CSTB	chr4	10077076	10078076	112	GGGTGCCGTAAGTTTCTATCAG	AAACAAGCCATTGCAACTATCC
ENSMODG00000021051	RUNX1	chr4	12639112	12640112	113	ACCAGAGTGATGTGGTGGAAG	AAAGAAGCAAGCACAGTTTTCC
ENSMODG00000007427	PDK3	chr4	41989413	41990413	114	AAAAACCCCTTTCCACAGTACA	AGATTGTGTCTTCTGCAAGCTG
ENSMODG00000001888	NFKBIZ	chr4	62096907	62097907	115	GCCAATTTCTCCATTCAGTTTC	GCATTGCCTCCTGAAAGAAATC
ENSMODG00000021670	SNORA2	chr4	62103338	62104338	116	ACCTGGAAATATGCCTCTCTGA	GTCTGTTGGCCATTCTAGATCC
ENSMODG00000018047	IFT57	chr4	67631607	67632607	117	GAGAGTCCCTCTGTTCTTCCAA	CTGAAGCAAGAAACAGTCCAAA
ENSMODG00000018216	GSK3B	chr4	83632774	83633774	118	TTCTTTGCCTTTCCAGATCACT	GTTTCACTCAGCAACACTGGTC
ENSMODG00000000944	UBXN4	chr4	132780390	132781390	119	CTAGAAAAACGTGGCGAAGACT	ATCTATGCGCACTGAGGAATCT
ENSMODG00000005628	IFIH1	chr4	168318450	168319450	120	TACTCATGGGGAATGTTCACTG	CTTTCCAGTCCTGGGGAACTAT
ENSMODG00000005665	GCA	chr4	168505310	168506310	121	TACAAAACCCTCACACAGTGGT	CAGCAAAGAAAATCTTGGGTCT
ENSMODG00000009617	MTX2	chr4	187738965	187739965	122	CTCTCAGGTTGGCCTAGAGAAA	GGAAGAGGGATCCATGGTAAAG
ENSMODG00000011664	GLS	chr4	208188190	208189190	123	CCTTCAGGAATACCAAGTCCAG	CTGGAAGGGTCCAAAGTTACAT
ENSMODG00000012380	C2orf69	chr4	221510565	221511565	124	CAGGGATTGCAGTTCATACTCA	GTGACAAGAATGAGGCACAAAA
ENSMODG00000014615	C11orf65	chr4	245943367	245944367	125	AGGATATAAGGTGGCCTCAACA	CAGCAATGTAATTTGGTTCCAG
ENSMODG00000000824	DCUN1D5	chr4	259665304	259666304	126	ACATACACACACACGCAGGTTT	AAGTCCGTCAAGTGTCATAGCA
ENSMODG00000004297	PICALM	chr4	325137648	325138648	127	AATCCAGTGGAAGAGAATGGAA	CTTGCAAAATCTAGGCACAGAA
ENSMODG00000004498	PCF11	chr4	328701595	328702595	128	TGCAAGTTTGTCCTGTTGAGTT	GTACACCATCTCCCAGCAAGAC
ENSMODG00000012783	ID3	chr4	359890347	359891347	129	CTCCTCAACAAAACAAAACACG	GCTGACTTTCTTTGGTGGAATC
ENSMODG00000013020	XM_001362581.1	chr4	360458330	360459330	130	TAGCTACAAAACAGCACGCATT	GGCCACTAATGGCATAACCATA
ENSMODG00000023715		chr4	385763442	385764442	131	CACTGCGGACAAATACCTACAA	CCCTAGTCATTCTAGCCCAGTG
ENSMODG00000009049	TMEM160	chr4	386060673	386061673	132	AGCTGGAGCTGGAGATGGAG	CTGGAAAGGAGGGGTGATTT
ENSMODG00000002366		chr4	387524083	387525083	133	AACTGCCAAATCATGACTTCCT	GAGAAACAGAAGGGAAAAAGCA
ENSMODG00000006096	TNFRSF25	chr4	394682732	394683732	134	CAAAGACGGTCTGGGATTCTAC	AGCAATATGAGATGCTCAAACG
ENSMODG00000023559		chr4	401589273	401590273	135	ATCATGCACCTAACTTGTGCTC	CACATCAGAGAATTCACACTGGA
ENSMODG00000017226	PPIH	chr4	429135945	429136945	136	CCTAACAATAAGCCCAAACTGC	GAAGAAGGAACTGGGGAAAACT
ENSMODG00000012053	SEC31A	chr5	35181234	35182234	137	CAGCCTTGACAAGTTGAGTGAT	GTGCTCAAAGTGGTTCTTACCC
ENSMODG00000004762	CCDC110	chr5	79622982	79623982	138	TGAAGTTCAGCGTCAACAAGAT	TTGGGGGAACAAAGTACATAGA
ENSMODG00000020706	IGFBP7	chr5	168647869	168648869	139	AGTAAGGAAGATGCTGGGGAAT	TGCGTAGATGCTAGGGATACAA
ENSMODG00000020677	TMEM165	chr5	170377569	170378569	140	GGCAAGGAAAACAAAAGTCCTA	GGGATGAAACCATAGGTGAACA
ENSMODG00000000034	PCDH7	chr5	195763940	195764940	141	GTTCCTGCAACCCAACATACTT	ACCACCAGCTAACACTTTTGTG
ENSMODG00000000423	SEPSECS	chr5	203290281	203291281	142	AGTGATGGTGTCTCTGCAGTTG	ATAAGGAAACTAAGGCCCATGA
ENSMODG00000006522	LDHA_MONDO	chr5	242663942	242664942	143	TGATCACCCTAGTGCAAATGTT	ACAACAATCCAACCAATTCCTC
ENSMODG00000007335	COPB1	chr5	247048855	247049855	144	TGCAAATAGCTCACTGGCTTTA	ATTGCACCCTTTCACATTCTCT
ENSMODG00000008096	ADM	chr5	250096219	250097219	145	GAACCTGGCTCACCAGATCTAC	AACACCAAACTTGACAGCAAGA
ENSMODG00000026540	U6	chr5	250107281	250108281	146	AAAGAAAGAAAGAAGCCCAACC	CTCCCTCGTCTATTCCCTTTTC
ENSMODG00000009646	WT1	chr5	250270154	250271154	147	AGGAAACGATATCCGAAAGACA	AGAGAGCTCAAGGACTGGACTG

ENSMODG00000013386	NXNL2	chr6	2293076	2294076	148	ATACTGTATTGGGCTCCTCTGG	TTCTGGCTGAATTTGGAGAGAT
ENSMODG00000003495	GAS1	chr6	15524842	15525842	149	CAAAGAACGCACTCATCTGAAC	AGACTCGCAGTACGAGGAGGAT
ENSMODG00000003820	CD2A1_MONDO	chr6	36296449	36297449	150	ACCATTTGGGGGTTTTCTTAGT	CCCAGGATCAGCTTCAATACAT
ENSMODG00000025162	XM_001373914.1	chr6	36333515	36334515	151	ATATTCTCCTCCCCTCTGCTTC	GGTTTCCTGGACACTCTGATCT
ENSMODG00000005539	UGCG	chr6	54243962	54244962	152	GGGAAGGAAGGGTTAAGCTACA	GCAGAGGAAATCCTTGACGTAT
ENSMODG00000008046	CYTH3	chr6	62389852	62390852	153	ATAGCACTTTGCTGTTTGAGCA	CTTTTCCAAAGCCCTTACCTTT
ENSMODG00000002641	GDA	chr6	94039653	94040653	154	CCTACTTGTGCCATGTAAGTGC	AAGTGGTTCCATTTTCAAGCTC
ENSMODG00000003635		chr6	102275090	102276090	155	GGAATAAGGAAGTCACCACTGC	ACAAGGAAAAGCTCGAATCAAC
ENSMODG00000004516	BHLHE40	chr6	236726356	236727356	156	CAGAGAAGATCCCTCCTCCTTT	GGGCTACTTTACAGCTTTTGGA
ENSMODG00000004830	MTMR14	chr6	242565134	242566134	157	AGGTTTTCAGCACCAACTGAAC	ATCTGGCTCAGCCTTACTTCAC
ENSMODG00000004766	COL4A1	chr7	82619875	82620875	158	CGACTTTGAAAGCAGGAGAACT	CTGATTTGCCTAATTGCTGACA
ENSMODG00000015025	CLK1	chr7	171107979	171108979	159	GTCTTGAGGCAACTTGAACCTC	ATTCTCAAGATGCTGACCATGA
ENSMODG00000015605	FN1	chr7	178177078	178178078	160	TCAGTTGCTGATTGTCCATCTT	ATCACCAACCATCATTACACCA
ENSMODG00000015703	CCNL1	chr7	244712188	244713188	161	ACGGCCACAAGAGGAAAAAGT	CCAAGACAGGAGTCAAGATTCG
ENSMODG00000014421	KLHDC5	chr8	25879609	25880609	162	ATGGTACAGGCTTGGACACTTT	CAGAGAGTTGGGAGCCATTTAG
ENSMODG00000020516	DUSP6	chr8	62627976	62628976	163	TGTAATGGAGTGATACGCTTGG	AAATCCAATATCTCCCCCAACT
ENSMODG00000017458	RASK_MONDO	chr8	93333479	93334479	164	TATTTCAGGGTGTTGATGATGC	GCCCTTAACTATTTTCCACTGC
ENSMODG00000018312	CCND2	chr8	114355198	114356198	165	GACTTCTCGAGAGCCAAAGAAC	TCTTCTAGGATTGCCTGAAAGC
ENSMODG00000018401	WNK1	chr8	117841274	117842274	166	CACTCAGTGGAGTGGAACAAGT	CACATCAAAACTGATGGCAAAG
ENSMODG00000018521	SLC38A2	chr8	122746963	122747963	167	CTGGCATAACAGCAAACTTGAA	CAGATTTTAGGGGGTTACAGGA
ENSMODG00000016607		chr8	126406300	126407300	168	ACCTTACCAAATACCCAGATGC	TACCACATTGCTTTTCATCACC
ENSMODG00000014499	COPG2	chr8	190464437	190465437	169	ATGAAGGCAAGGTACTCTCAGC	TAGATTGGCCTTAGGAGATGGA
ENSMODG00000013750	C7orf55	chr8	201464609	201465609	170	CACTTCCAAGCTGCTACCTACC	CCATGTTCTTCCTAGCTGTGTG
ENSMODG00000013737	KLRG2	chr8	201570807	201571807	171	CCTTTTGGATCTGCTCAAGAAC	AGGCTCAAGGCTTTAGAATGTG
ENSMODG00000006521	PFKP	chr8	232504676	232505676	172	ATGGTGGTTGAAACTACGACCT	ACTGAGAGCTCTGGAATCTGCT
ENSMODG00000008021	BAMBI	chr8	244170301	244171301	173	CTGGAGAGAGGAAAGAAGGCTA	GGCAAAGTTAGACTTGGAATGC
ENSMODG00000009719	STAM	chr8	256334468	256335468	174	CGCAGAAAGCCATGATACATT	TGCTGCTACTGACGTCACAATA
ENSMODG00000009818	VIM	chr8	256730655	256731655	175	CTGAAAATTGCAGTTTGACCAC	CAACACCATGATGATCTGGAA
ENSMODG00000023924	TWIST1	chr8	304139473	304140473	176	CATCGACTTCCTCTACCAGGTC	GGTGGAATTTGGGAGTTTACAA
ENSMODG00000021336	NDUFV1	chrUn	7775211	7776211	177	CATCAAGTTCGACCTGAACAAG	ACTAATCACGGTTGGGGAATGT
ENSMODG00000020581	ANKFY1	chrUn	38479834	38480834	178	ATTAAACCCCATGAGGAAAACC	GCGTTGGTCTGAGTGAATAAAG
ENSMODG00000007100	IGF2R	chr2	442547176	442548176	179	GGGAGAGAAGGAGAGAAAAGGA	GAAGCACCCGAGAAACTAAAGA

Supplemental Table S4. SNP variation detected between individuals A0563 (LL1) X A0573 (LL2). 42 total SNPs. Genes chosen for SNP confirmation are indicated by an asterisk.

Chromosome	Start	End	Ensembl Gene ID	SNP/Genotype A0563	A0573	POSITION
CHR1	50888330	50889340	ENSMODG00000000633	A	AG	50888587*
CHR1	108942890	108943900	ENSMODG00000011165	G	AG	108943512
CHR1	148353080	148354090	ENSMODG00000011135	A	AG	148353432
CHR1	285526640	285527650	ENSMODG00000012905	T	CT	285527301*
CHR1	285630810	285631820	ENSMODG00000012822	C	CT	285631226
CHR1	333227660	333228670	ENSMODG00000010724	C	CT	333227821*
CHR1	333244980	333245990	ENSMODG00000023761	AG	A	333245463*
CHR1	333338010	333339010	ENSMODG00000010717	G	C	333338396
CHR1	343914250	343915260	ENSMODG00000009436	C	GC	343915130*
CHR1	510096290	510097300	ENSMODG00000015575	A	AT	510096540
CHR1	589524310	589525320	ENSMODG00000008637	C	T	589524693
CHR1	624802970	624803980	ENSMODG00000003396	AG	A	624803564*
CHR1	717916460	717917470	ENSMODG00000015604	C	CT	717917106
CHR2	78706630	78707640	ENSMODG00000007194	G	AG	78707300
CHR2	169499700	169500710	ENSMODG00000014128	AG	A	169499823
CHR2	194012780	194013790	ENSMODG00000014859	A	AG	194013292
CHR2	275269390	275270390	ENSMODG00000013704	AG	G	275269467
CHR2	285218800	285219810	ENSMODG00000012785	C	A	285219179*
CHR2	302215600	302216610	ENSMODG00000018800	A	AC	285219179*
CHR2	307679000	307680010	ENSMODG00000018721	A	G	307679839*
CHR3	16801020	16802030	ENSMODG00000019470	AT	T	16801290*
CHR3	49543210	49543820	ENSMODG00000025244	A	AG	49543500*
CHR3	421671140	421672150	ENSMODG00000000768	G	GT	421671879
CHR3	437574710	437575720	ENSMODG00000011184	G	T	437574828*
CHR3	460433660	460434670	ENSMODG00000003841	T	CT	460434242*
CHR3	501254990	501256000	ENSMODG00000023660	A	AG	501255667
CHR4	12639110	12640120	ENSMODG00000021051	GT	G	12639255
CHR4	41989410	41990420	ENSMODG00000007427	T	A	41989927
CHR4	62096900	62097910	ENSMODG00000001888	G	C	62097383
CHR4	67631600	67632610	ENSMODG00000018047	G	T	67632141
CHR4	132780390	132781390	ENSMODG00000000944	G	AC	132780626
CHR4	168318450	168319450	ENSMODG00000005628	A	G	168319167
CHR4	259665300	259666310	ENSMODG00000000824	AG	G	259665471*
CHR5	242663940	242664950	ENSMODG00000006522	A	AT	242664163
CHR6	236726350	236727360	ENSMODG00000004516	G	AG	236726484
CHR6	242565130	242566140	ENSMODG00000004830	G	A	242565229
CHR7	244712180	244713190	ENSMODG00000015703	AC	C	244712322
CHR8	25879600	25880610	ENSMODG00000014421	C	CT	25879766
CHR8	62627970	62628980	ENSMODG00000020516	G	AG	62628747*
CHR8	201570800	201571810	ENSMODG00000013737	T	AG	201570943
CHR8	244170300	244171310	ENSMODG00000008021	C	T	244171254
CHR8	256730650	256731660	ENSMODG00000009818	A	AT	256731126*

Supplemental Table S5. SNP variation detected between individuals A0552 (LL1) X A0568 (LL2). 42 total SNPs. Genes chosen for SNP confirmation are indicated by an asterisk.

Chromosome	Start	End	Ensembl Gene ID	SNP/Genotype A0552	A0568	POSITION
CHR1	276674510	276675520	ENSMODG00000011684	AT	AT	276675391
CHR1	285526640	285527650	ENSMODG00000012905	T	CT	285527301*
CHR1	285630810	285631820	ENSMODG00000012822	A	AG	285631526
CHR1	333227660	333228670	ENSMODG00000010724	T	GT	333228053*
CHR1	333244980	333245990	ENSMODG00000023761	A	AG	333245463*
CHR1	333338010	333339010	ENSMODG00000010717	G	C	333338396
CHR1	343914250	343915260	ENSMODG00000009436	C	CG	343915130*
CHR1	663121130	663122140	ENSMODG00000008486	CT	C	663121514*
CHR2	109874510	109875520	ENSMODG00000001558	C	CT	109874653
CHR2	139634610	139635610	ENSMODG00000006246	C	G	139635294*
CHR2	194012780	194013790	ENSMODG00000014859	CT	T	194013302
CHR2	206114030	206115040	ENSMODG00000024021	AG	G	206114857
CHR2	225162270	225163280	ENSMODG00000004465	C	T	225162765
CHR2	275269390	275270390	ENSMODG00000013704	AG	G	275269467
CHR2	285218800	285219810	ENSMODG00000012785	AC	C	285219179*
CHR2	302215600	302216610	ENSMODG00000018800	G	GT	302216483*
CHR2	307679000	307680010	ENSMODG00000018721	A	G	307679357*
CHR2	332904140	332905150	ENSMODG00000018444	C	CT	332904537
CHR2	442547170	442548180	ENSMODG00000007100	A	A	442547809*
CHR2	459548410	459549420	ENSMODG00000004472	G	AG	459548616
CHR2	505025230	505026240	ENSMODG00000019221	G	A	505025646
CHR2	520272570	520273580	ENSMODG00000016394	G	CG	520270108
CHR3	49543210	49543820	ENSMODG00000025244	A	G	49543500*
CHR3	232703750	232704760	ENSMODG00000020286	G	AG	232704431
CHR3	269085270	269086280	ENSMODG00000021462	G	AG	269085368*
CHR3	377688000	377689010	ENSMODG00000004746	C	CT	377688211
CHR3	437574710	437575720	ENSMODG00000011184	G	GT	437574828*
CHR3	437738810	437739820	ENSMODG00000011203	G	AG	437739703
CHR3	460433660	460434670	ENSMODG00000003841	T	C	460434242*
CHR4	10077070	10078080	ENSMODG00000021035	T	GT	10077387*
CHR4	12639110	12640120	ENSMODG00000021051	GT	G	12639255
CHR4	41989410	41990420	ENSMODG00000007427	T	AT	41989927
CHR4	62096900	62097910	ENSMODG00000001888	CT	C	62097717
CHR4	67631600	67632610	ENSMODG00000018047	T	C	67631863
CHR4	168318450	168319450	ENSMODG00000005628	AG	A	168319167
CHR4	259665300	259666310	ENSMODG00000000824	AG	G	259665471*
CHR5	250270150	250271160	ENSMODG00000009646	AG	A	250270818*
CHR6	2293070	2294080	ENSMODG00000013386	C	CT	2293331
CHR6	94039650	94040660	ENSMODG00000002641	AG	G	94040117
CHR8	25879600	25880610	ENSMODG00000014421	C	T	25879766
CHR8	62627970	62628980	ENSMODG00000020516	CT	T	62628670*
CHR8	256730650	256731660	ENSMODG00000009818	A	AT	256731126*

Supplemental Table S6. SNP variation detected between individuals A0567 (LL2) X A0578 (LL1). 49 total SNPs. Genes chosen for SNP confirmation are indicated by an asterisk.

Chromosome	Start	End	Ensembl Gene ID	SNP/Genotype A0567	A0578	POSITION
CHR1	50888330	50889340	ENSMODG00000000633	G	AG	50888812*
CHR1	108942890	108943900	ENSMODG00000011165	A	AG	108943512
CHR1	148353080	148354090	ENSMODG00000011135	AT	T	148354005
CHR1	276674510	276675520	ENSMODG00000011684	T	AT	276675391
CHR1	285526640	285527650	ENSMODG00000012905	T	CT	285527301*
CHR1	285630810	285631820	ENSMODG00000012822	A	AG	285631526
CHR1	333227660	333228670	ENSMODG00000010724	GT	T	333228053*
CHR1	333244980	333245990	ENSMODG00000023761	AG	A	333245463*
CHR1	333338010	333339010	ENSMODG00000010717	C	G	333338396
CHR1	343914250	343915260	ENSMODG00000009436	C	CG	343915130*
CHR1	401900280	401901290	ENSMODG00000019496	C	T	401900959
CHR1	589524310	589525320	ENSMODG00000008637	C	CT	589524693
CHR1	624802970	624803980	ENSMODG00000003396	A	AG	624803564*
CHR1	663121130	663122140	ENSMODG00000008486	C	CT	663121514*
CHR2	57215440	57216440	ENSMODG00000001321	CT	T	57215808
CHR2	78706630	78707640	ENSMODG00000007194	AG	A	78706823
CHR2	109874510	109875520	ENSMODG00000001558	C	CT	109874653
CHR2	139634610	139635610	ENSMODG00000006246	G	C	139635294*
CHR2	206114030	206115040	ENSMODG00000024021	G	AG	206114857
CHR2	275269390	275270390	ENSMODG00000013704	G	AG	275269467
CHR2	285218800	285219810	ENSMODG00000012785	A	AC	285219179*
CHR2	307679000	307680010	ENSMODG00000018721	G	A	307679839*
CHR2	442547170	442548180	ENSMODG00000007100	A	C	442547809*
CHR2	459548410	459549420	ENSMODG00000004472	A	AG	459549205
CHR2	505025230	505026240	ENSMODG00000019221	A	AG	505025646
CHR2	522386350	522387360	ENSMODG00000023140	CT	C	522386432
CHR3	16801020	16802030	ENSMODG00000019470	T	G	16801298*
CHR3	49543210	49543820	ENSMODG00000025244	AG	AG	49543500*
CHR3	269085270	269086280	ENSMODG00000021462	AG	G	269085368*
CHR3	377688000	377689010	ENSMODG00000004746	C	CT	377688068
CHR3	437574710	437575720	ENSMODG00000011184	GT	G	437574828*
CHR3	437738810	437739820	ENSMODG00000011203	AG	G	437739703
CHR3	443006000	443007010	ENSMODG00000001178	T	CT	443006178
CHR3	460433660	460434670	ENSMODG00000003841	C	T	460434242*
CHR3	499708700	499709710	ENSMODG00000025637	AG	A	499709016
CHR4	10077070	10078080	ENSMODG00000021035	GT	T	10077387*
CHR4	12639110	12640120	ENSMODG00000021051	G	GT	12639255
CHR4	41989410	41990420	ENSMODG00000007427	A	T	41989927
CHR4	67631600	67632610	ENSMODG00000018047	CT	C	67632076
CHR4	221510560	221511570	ENSMODG00000012380	AG	A	221511019
CHR4	259665300	259666310	ENSMODG00000000824	G	AG	259665471*
CHR5	250270150	250271160	ENSMODG00000009646	T	CT	250270554*
CHR6	2293070	2294080	ENSMODG00000013386	CT	C	2293331
CHR6	54243960	54244970	ENSMODG00000005539	T	C	54244551*
CHR8	25879600	25880610	ENSMODG00000014421	T	CT	25879766
CHR8	62627970	62628980	ENSMODG00000020516	CT	T	62628207*
CHR8	201570800	201571810	ENSMODG00000013737	G	AG	201571369
CHR8	232504670	232505680	ENSMODG00000006521	A	AC	232505189
CHR8	256730650	256731660	ENSMODG00000009818	T	AT	256731126*

Supplemental Table S7. SNP variation detected between individuals A0566 (LL2) X A0549 (LL1). 38 total SNPs. Genes chosen for SNP confirmation are indicated by an asterisk.

Chromosome	Start	End	Ensembl Gene ID	SNP/Genotype A0566	A0549	POSITION
CHR1	50888330	50889340	ENSMODG00000000633	AG	G	50888812*
CHR1	285526640	285527650	ENSMODG00000012905	CT	T	285527301*
CHR1	285630810	285631820	ENSMODG00000012822	CT	C	285631226
CHR1	333227660	333228670	ENSMODG00000010724	GT	GT	333228053*
CHR1	343914250	343915260	ENSMODG00000009436	G	CG	343915130*
CHR1	624802970	624803980	ENSMODG00000003396	A	G	624803564*
CHR1	663121130	663122140	ENSMODG00000008486	C	CT	663121514*
CHR2	139634610	139635610	ENSMODG00000006246	CG	C	139635294*
CHR2	275269390	275270390	ENSMODG00000013704	CT	C	275269633
CHR2	285218800	285219810	ENSMODG00000012785	C	AC	285219179*
CHR2	302215600	302216610	ENSMODG00000018800	T	G	302216483*
CHR2	307679000	307680010	ENSMODG00000018721	G	AG	307679357*
CHR2	332904140	332905150	ENSMODG00000018444	AG	G	332904873
CHR2	505025230	505026240	ENSMODG00000019221	A	AG	505025646
CHR2	522386350	522387360	ENSMODG00000023140	T	C	522386432
CHR3	16801020	16802030	ENSMODG00000019470	A	AT	16801605*
CHR3	49543210	49543820	ENSMODG00000025244	G	AG	49543590*
CHR3	377688000	377689010	ENSMODG00000004746	CT	C	377688211
CHR3	421671140	421672150	ENSMODG00000000768	GT	GT	421671879
CHR3	443006000	443007010	ENSMODG00000001178	CT	T	443006178
CHR3	460433660	460434670	ENSMODG00000003841	C	CT	460434242*
CHR3	499708700	499709710	ENSMODG00000025637	G	AG	499709016
CHR4	10077070	10078080	ENSMODG00000021035	G	T	10077387*
CHR4	41989410	41990420	ENSMODG00000007427	A	T	41989927
CHR4	67631600	67632610	ENSMODG00000018047	CT	C	67632076
CHR4	132780390	132781390	ENSMODG00000000944	AC	C	132780626
CHR4	187738960	187739970	ENSMODG00000009617	CT	T	187739279
CHR4	259665300	259666310	ENSMODG00000000824	G	AG	259665471*
CHR4	360458330	360459330	ENSMODG00000013020	A	AT	360458582
CHR5	242663940	242664950	ENSMODG00000006522	AT	A	242664163
CHR5	250270150	250271160	ENSMODG00000009646	A	A	250270818*
CHR6	2293070	2294080	ENSMODG00000013386	C	AC	2293370
CHR6	36333510	36334520	ENSMODG00000025162	AG	A	36333856*
CHR6	54243960	54244970	ENSMODG00000005539	CT	T	54244551*
CHR6	94039650	94040660	ENSMODG00000002641	G	AG	94040117
CHR7	178177070	178178080	ENSMODG00000015605	CT	C	178177875
CHR7	62627970	62628980	ENSMODG00000020516	CT	T	62628207*
CHR7	201570800	201571810	ENSMODG00000013737	AT	T	201570943

Supplemental Table S8. Summary of Pyrosequencing results for *Meis1*, *Cstb*, and *Rpl17*. Animal ID and cross type are indicated (1 - A0xxx - LL1 X LL2 and 2 - A0xxx- LL2 X LL1). The genotypes of the offspring are shown with the maternal allele listed first for all animals. Reference (Ref.) and alternative (Alt.) alleles and their respective expression percentages are shown in the subsequent columns. SNPs identified in Supplemental Figure F3 were used to assay allele specific expression for *Meis1* and *Cstb*. Genotypes for *Rpl17* were inferred from the PCR-Seq data due to the lack of quality Sanger reads for the gDNA. NM – Not measured

Animal ID	Cross Type	Gene	Genotype	Ref. Allele	Alter. Allele	% Ref Allele	% Alt Allele
A0690	1	*Meis1*	A/A	A	G	NM	NM
A0694	1	*Meis1*	G/G	A	G	0.0%	100.0%
A0695	1	*Meis1*	G/A	A	G	23.1%	76.9%
A0719	2	*Meis1*	A/G	A	G	74.8%	25.2%
A0727	2	*Meis1*	A/G	A	G	93.2%	6.8%
A0690	1	*Cstb*	T/G	T	G	0.0%	100.0%
A0694	1	*Cstb*	G/T	T	G	0.0%	100.0%
A0695	1	*Cstb*	T/T	T	G	100.0%	0.0%
A0719	2	*Cstb*	G/T	T	G	1.6%	98.4%
A0727	2	*Cstb*	G/T	T	G	15.6%	84.4%
A0690	1	*Rpl17*	G/T	T	G	0.0%	100.0%
A0694	1	*Rpl17*	G/T	T	G	0.0%	100.0%
A0695	1	*Rpl17*	G/T	T	G	0.0%	100.0%
A0719	2	*Rpl17*	G/T	T	G	0.0%	100.0%

Supplemental Table S9. Bisulfite PCR primers for *Meis*, *Cstb*, *Rpl17*, and *Igf2r*. Primers designed using Methyl Primer Express Software (Applied Biosystems, Inc.) to target the promoter CpG islands in bisulfite treated DNA. Two primers produced amplicons for *Cstb*. For *Igf2r*, we designed two primers each for the promoter CpG island and the CpG island at intron 11.

Gene	CpG Island Location	Forward Primer	Reverse Primers	Amplicon Size (bp)
Meis1	chr1:624957358-624957620	GATTTAGGGTTGGAGAAAGTTAG	CAAAAAAAAAAAAAAATCCCTCT	205
Cstb_1	chr4:10081961-10082165	ATTTATTGTTTAAAAGTGGGAGG	AAAAACAAAAAACTCAAATTTCC	274
Cstb_2	chr4:10081961-10082165	ATGGAAGGAAGGAGTTTAGTT	AAATTCTTATCTTAAAAAAATCAACCT	274
Rpl17	chr3:437577629-437578003	GGAAAAAGTTTTTGGAAATTGT	AAAATTAACCAAATAACAACCCC	175
Igf2r_Promoter	chr2:442405660-442406525	ATATTGGTTATAGGGATAAGGTTAGG	CATAAACTTCCCAAAATACTTCAC	283
Igf2r_Promoter	chr2:442405660-442406525	TTTGAGATGAGTGTTAGAAAATT	AACTAATAACCCCTAATCCATAA	157
Igf2r - Intron 11	chr2:442405660-442406525	AAGTGGTAAAAGGTTTTTTAATGTT	AAATCTTTAATCATTTCCTCCC	224
Igf2r - Intron 11	chr2:442405660-442406525	TTTATTTAGTTAAATTGTTTGGAAGAA	AAAAAAACCCAATAAAAAAACC	161
