# Supplementary material for: Genome-wide histone state profiling of fibroblasts from the opossum, Monodelphis domestica, identifies the first marsupial-specific imprinted gene
Source: BMC Genomics. 2014 Jan 31;15:89. doi: 10.1186/1471-2164-15-89 (PMC3912494; doi:10.1186/1471-2164-15-89)

Additional File 2

Supplemental Figures

Supplemental Figure S1. Pedigree Information. A) Crosses and animals used for ChIP-seq experiments. Animal IDs and stock source (LL1 or LL2) are indicated. B) Crosses and animals used for DNA and RNA verification experiments. Animal IDs are indicated. Top Panel: LL1 females crossed with LL2 males. Bottom Panel: LL2 females crossed with LL1 males.

A)

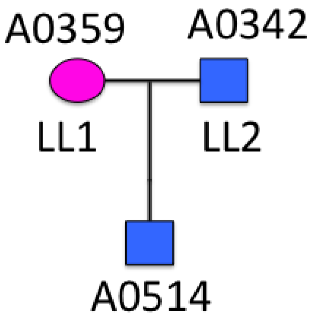

B)

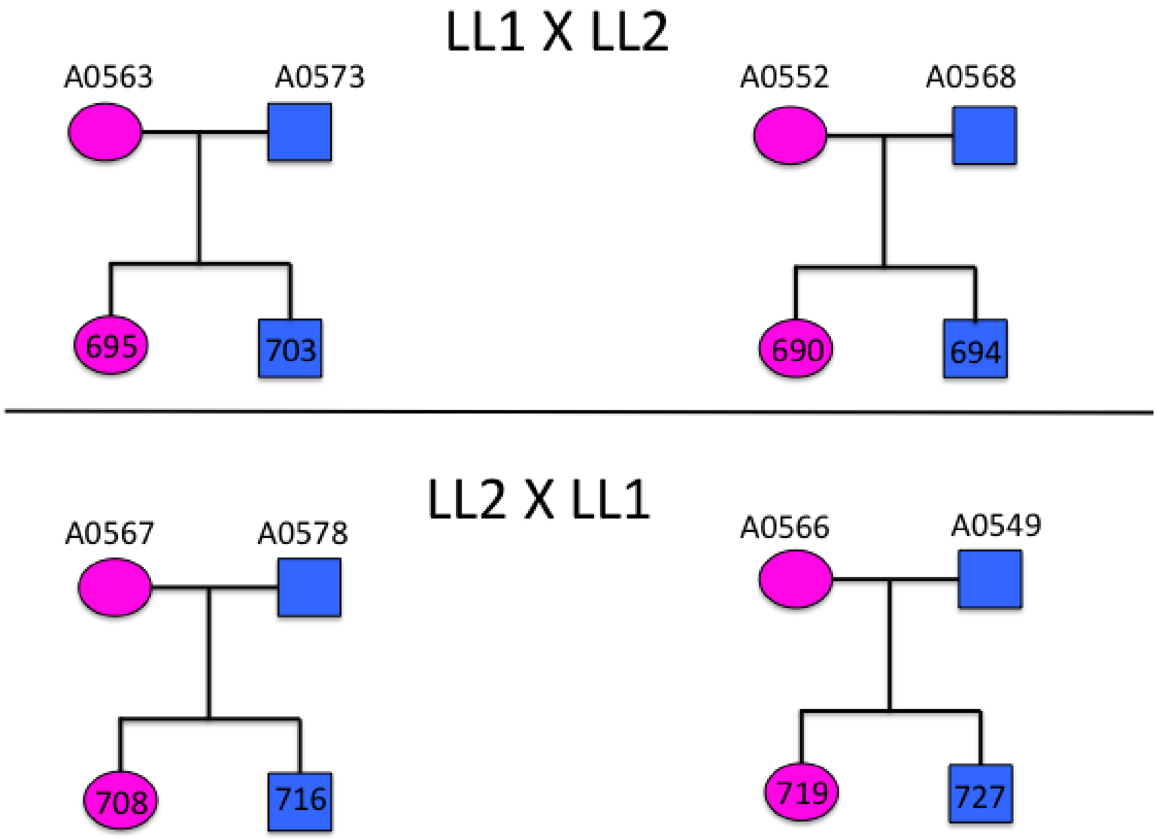

Supplemental Figure S2. Histone modification profiles for *Cstb* and *Rpl17*. Green panels = ChIP-seq raw read alignments for H3K4me3 (top) and H3K9Ac (bottom). Red panels = ChIP-seq raw read alignments for H3K9me3 (top) and H3K27me3 (bottom). Black panel = input. Blue bars above ChIP-seq panels are areas of significant enrichment determined by MACS ($p \leq 10^{-5}$). Blue bar in bottom panel represents the gene annotation with the direction of transcription indicated by the black arrows. Annotated CpG islands are indicated by black bars one panel above the annotation.

A)

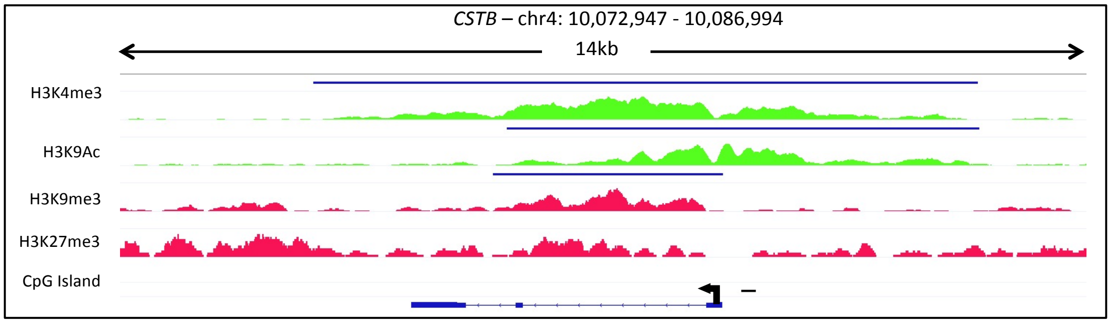

B)

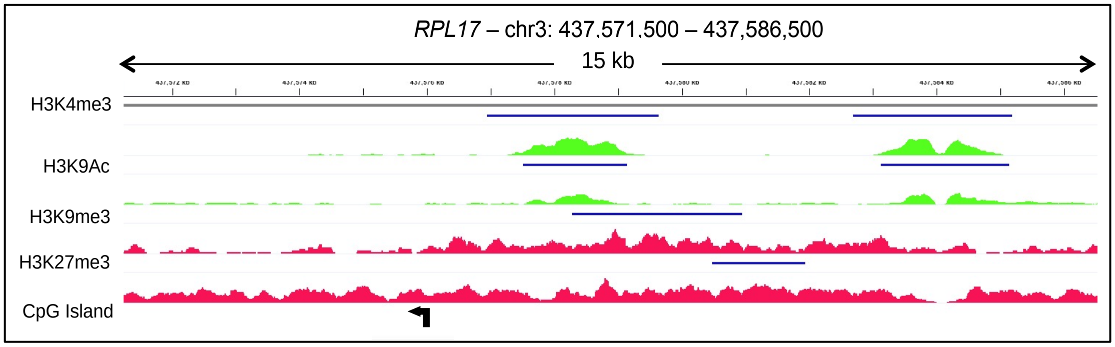

Supplemental Figure S3. Pedigrees and Sanger Sequencing Results for A) *Meis1*, B) *Cstb*, C) *Rpl17*, and D) *Igf2r* from reciprocal crosses. Animal IDs are presented as A0xxx, Sanger sequences generated from cDNA (top) and gDNA (bottom) are shown for informative animals. Black arrows indicate positions of informative SNPs. Only cDNA data is shown for *Rpl17*. Lower case letters for *Meis1* indicate leaky expression of the imprinted allele. Sanger data are not definitive for gene expression and were used exclusively to screen for probable heterozygotes for pyromark analysis of relative allelic expression.

A)

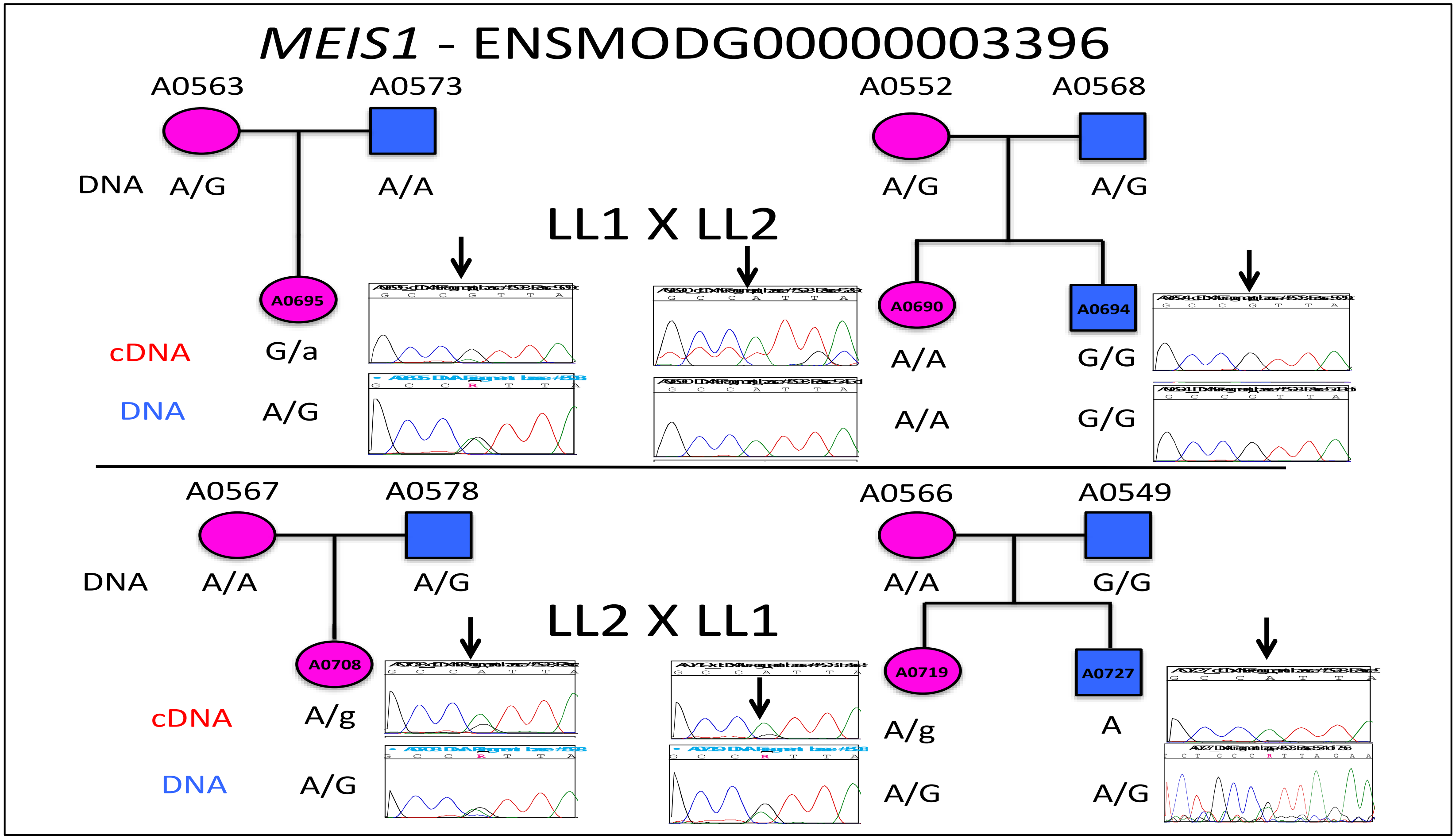

B)

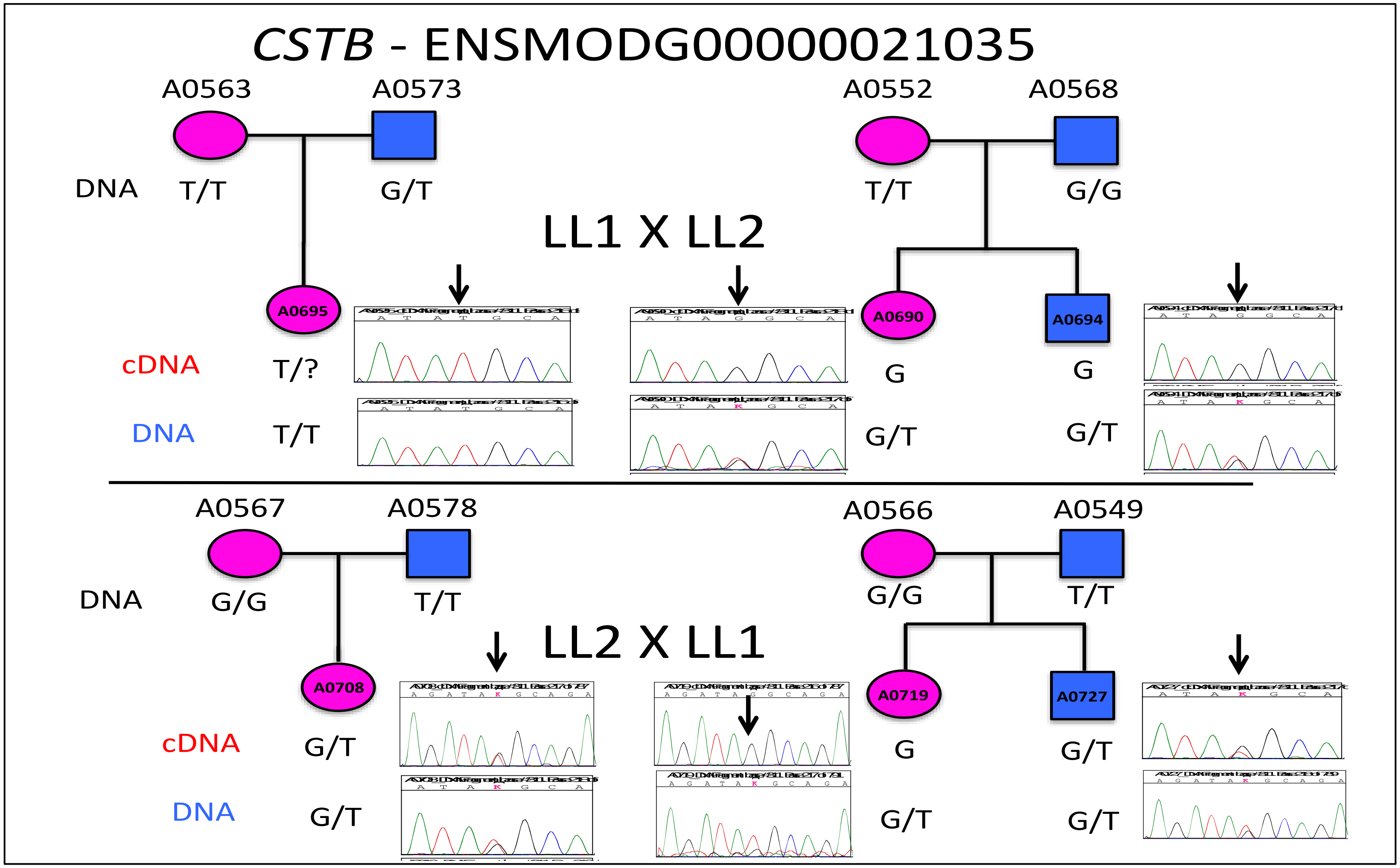

CSTB - ENSMODG00000021035
A0563
A0573
A0552
A0568
DNA
T/T
G/T
T/T
G/G
LL1 X LL2
A0695
A0690
A0694
cDNA
T/?
G
G
DNA
T/T
G/T
G/T
A0567
A0578
A0566
A0549
DNA
G/G
T/T
G/G
T/T
LL2 X LL1
A0708
A0719
A0727
cDNA
G/T
G
G/T
DNA
G/T
G/T
G/T

C)

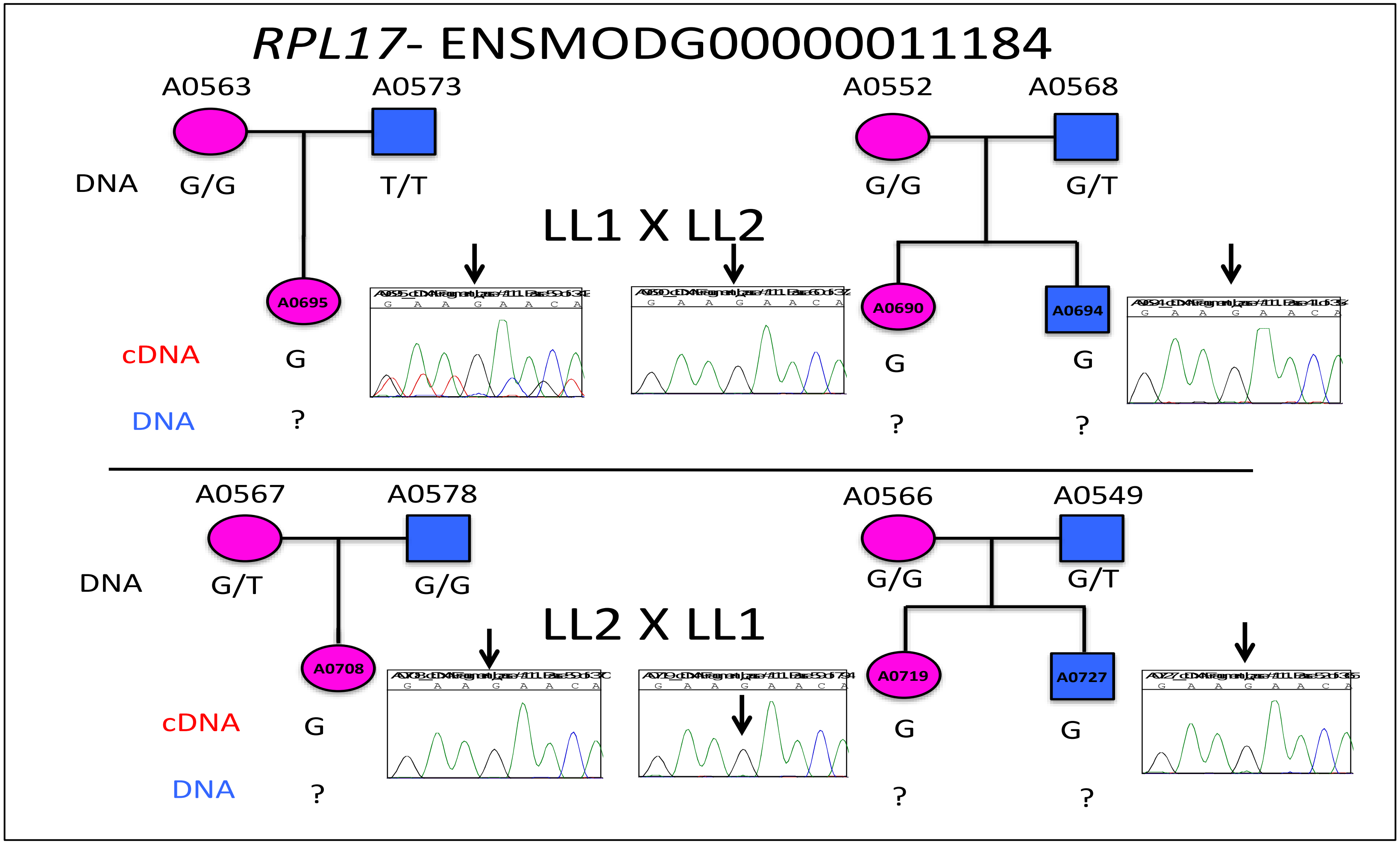

RPL17- ENSMODG00000011184
A0563
A0573
A0552
A0568
DNA
G/G
T/T
G/G
G/T
LL1 X LL2
A0695
A0690
A0694
cDNA
G
G
G
DNA
?
?
?
A0567
A0578
A0566
A0549
DNA
G/T
G/G
G/G
G/T
LL2 X LL1
A0708
A0719
A0727
cDNA
G
G
G
DNA
?
?
?

D)

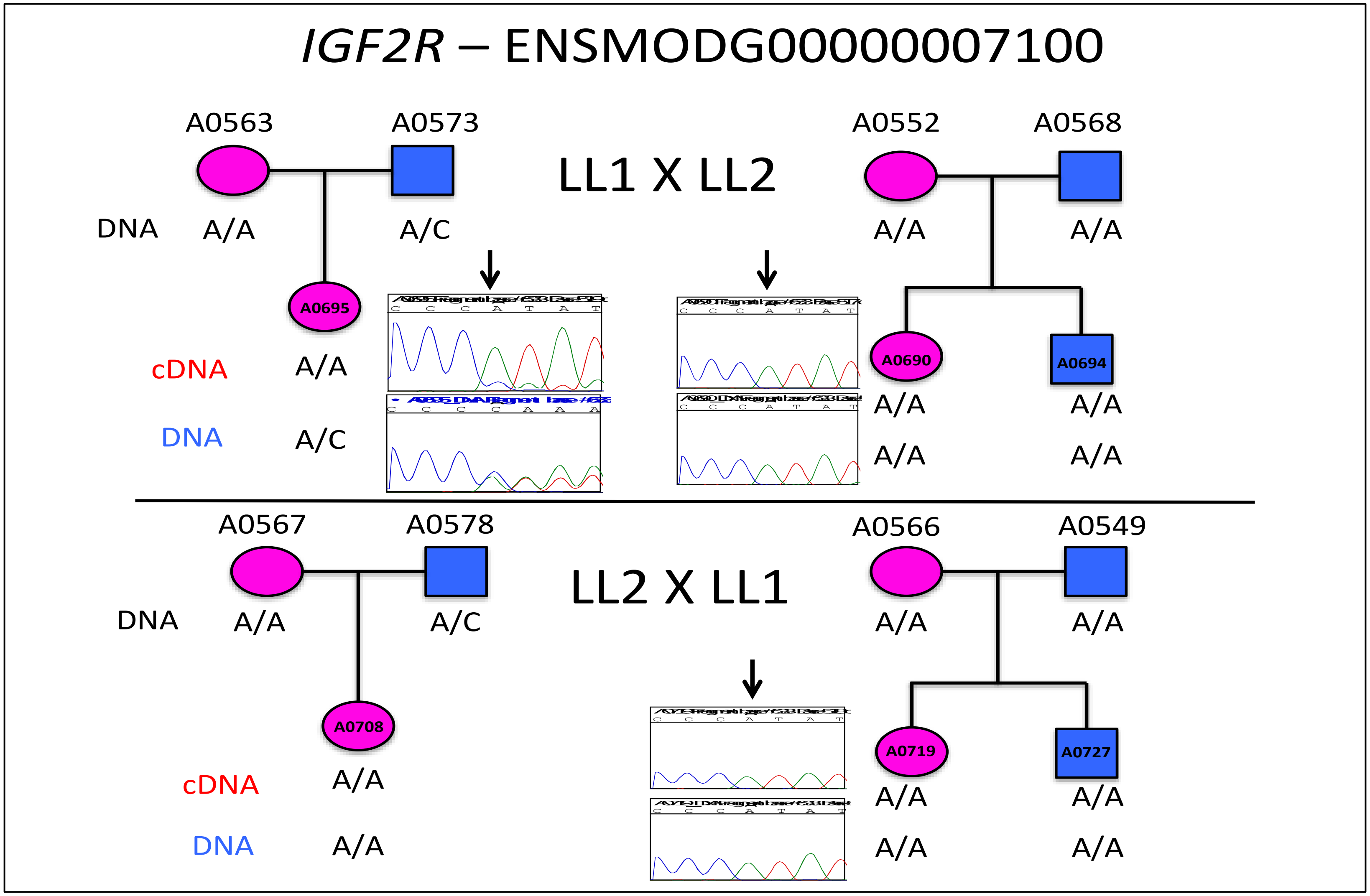
IGF2R – ENSMODG00000007100
A0563
A0573
A0552
A0568
LL1 X LL2
DNA
A/A
A/C
A/A
A/A
A0695
A0690
A0694
cDNA
A/A
A/A
A/A
DNA
A/C
A/A
A/A
A0567
A0578
A0566
A0549
LL2 X LL1
DNA
A/A
A/C
A/A
A/A
A0708
A0719
A0727
cDNA
A/A
A/A
A/A
DNA
A/A
A/A
A/A

Supplemental Figure S4. Histone modification profiles and DNA methylation at *Igf2r*. Promoter, CpG island hypomethylation, and a differentially methylated region at the CpG island at intron 11 for animal A0694. Top two panels are the histone modifications, H3K4me3 and H3K9Ac and significant peaks as called by MACS ($p \leq 10^{-5}$).

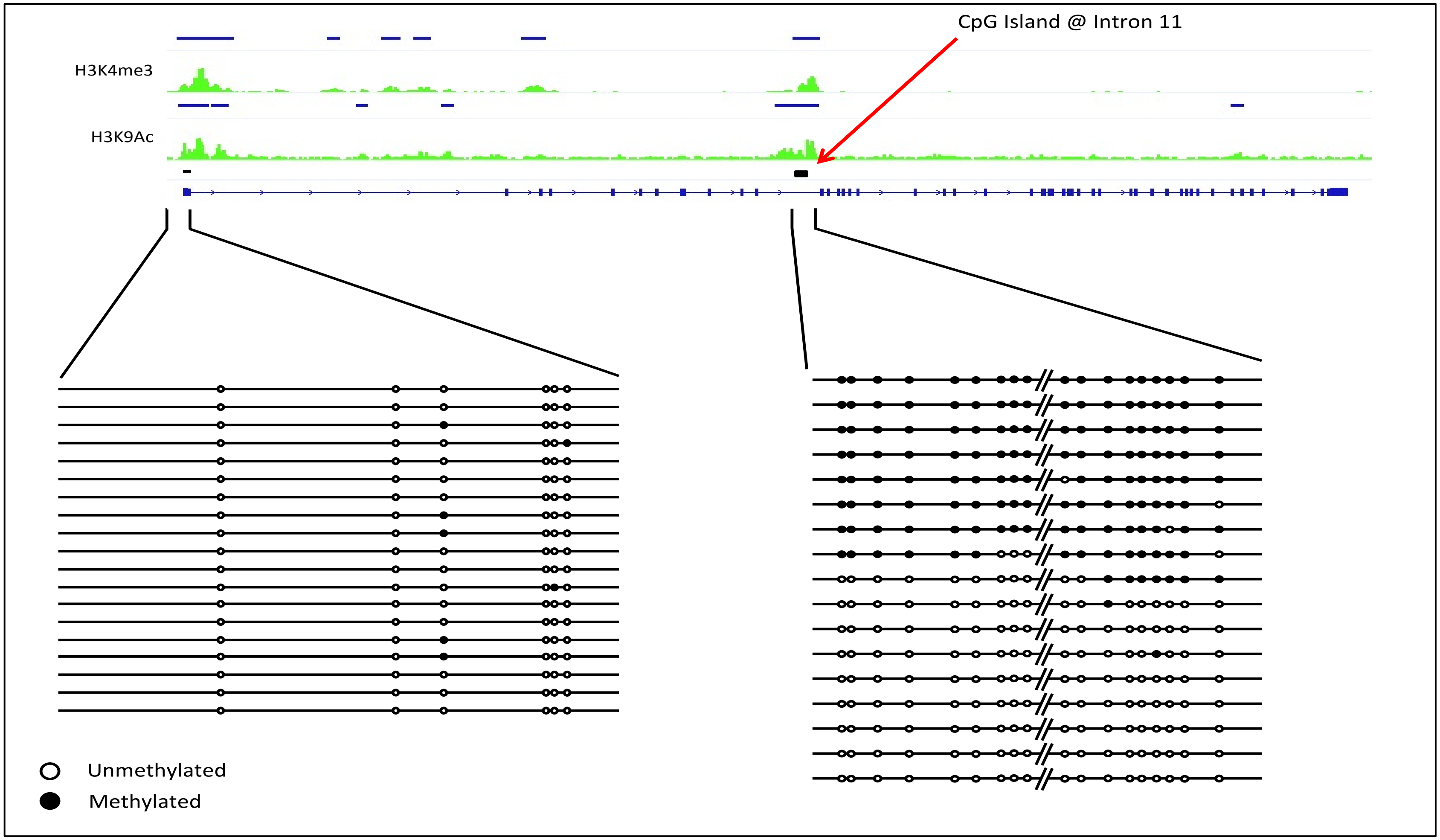

Supplemental Figure S5. Histone modification profiles at A) *Htr2a*, B) *L3mbtl*, and C) *Mest*. Green panels = ChIP-seq raw read alignments for H3K4me3 (top) and H3K9Ac (bottom). Red panels = ChIP-seq raw read alignments for H3K9me3 (top) and H3K27me3 (bottom). Black panel = input. Blue bars above ChIP-seq panels are areas of significant enrichment determined by MACS ($p \leq 10^{-5}$). Blue bar in bottom panel represents the gene annotation with the direction of transcription indicated by the black arrow. Annotated CpG islands are indicated by black bars one panel above the annotation. Gaps in the genome assembly are in the panel above the CpG Island panel (only seen in B).

A) *Htr2a*

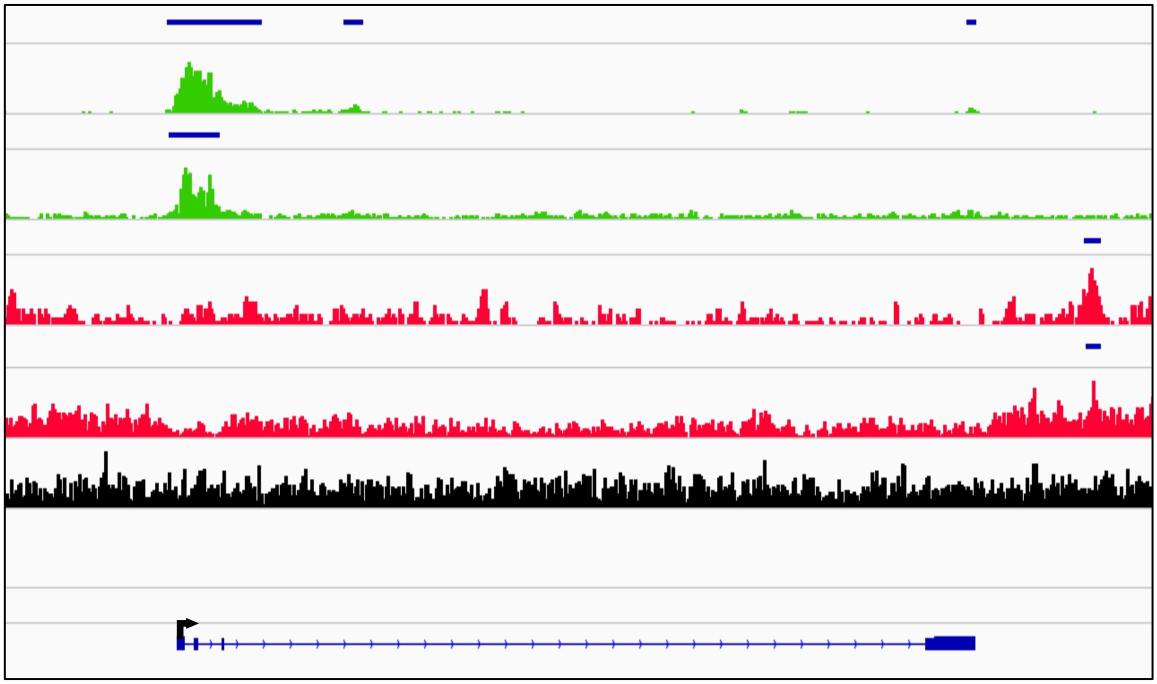

B) *L3mbtl*

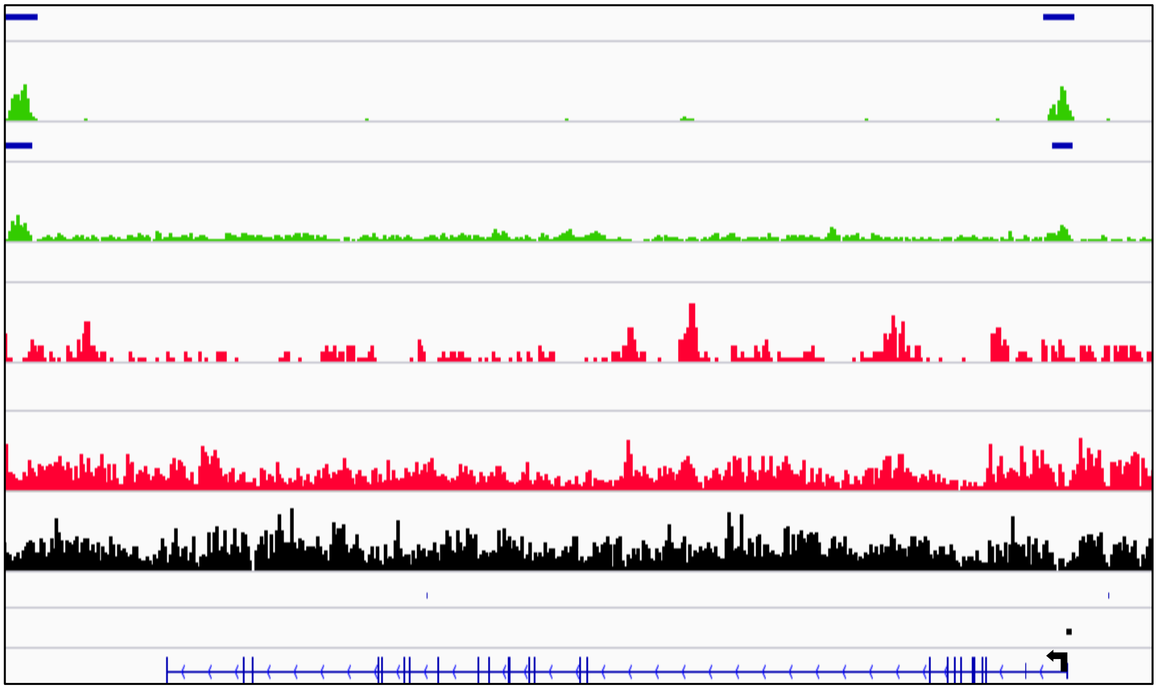

C) *Mest*

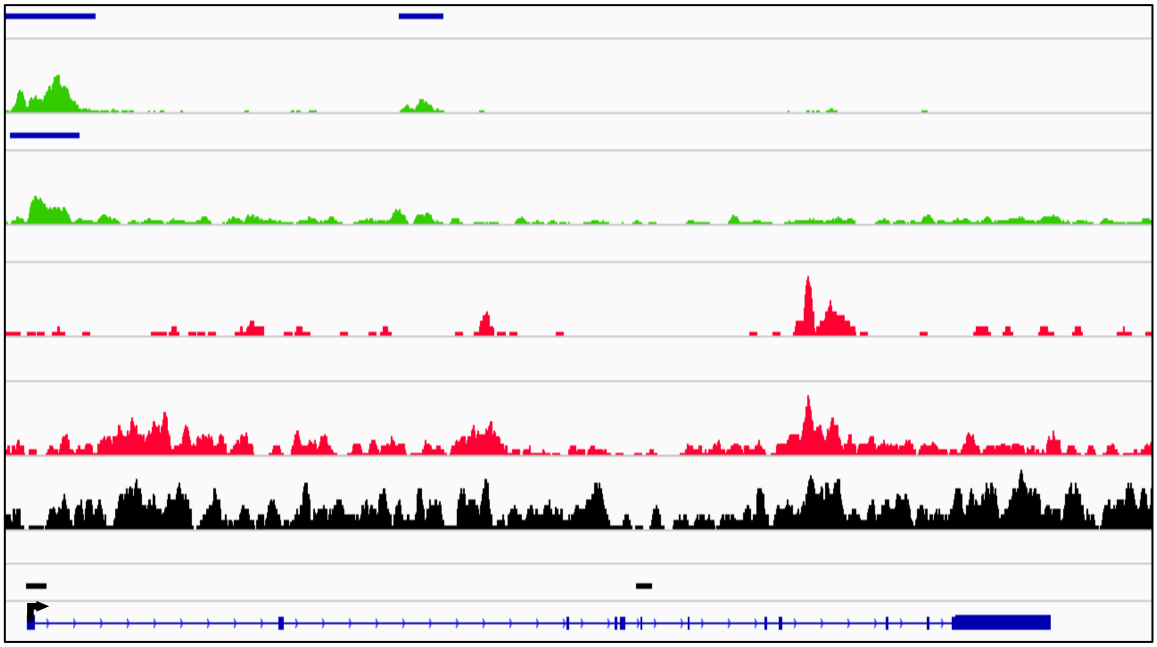

Supplement: Additional file 2 — Supplemental Figures. [file 1471-2164-15-89-S2.pdf]
